# TERMINAL FLOWER‐1/CENTRORADIALIS inhibits tuberisation via protein interaction with the tuberigen activation complex

**DOI:** 10.1111/tpj.14898

**Published:** 2020-07-14

**Authors:** Xing Zhang, Raymond Campbell, Laurence J. M. Ducreux, Jennifer Morris, Pete E. Hedley, Elena Mellado‐Ortega, Alison G. Roberts, Jennifer Stephens, Glenn J. Bryan, Lesley Torrance, Sean N. Chapman, Salomé Prat, Mark A. Taylor

**Affiliations:** ^1^ College of Life Science and Technology Huazhong Agricultural University Wuhan 430070 China; ^2^ Cell and Molecular Sciences The James Hutton Institute Invergowrie, Dundee DD2 5DA UK; ^3^ School of Biology Biomolecular Sciences Building University of St Andrews North Haugh St Andrews Fife Y16 9ST UK; ^4^ Centro Nacional de Biotecnología C/Darwin no. 3, Campus de Cantoblanco Madrid 28049 Spain

**Keywords:** protein–protein interaction, *Solanum tuberosum* (potato), *TERMINAL FLOWER 1/CENTRORADIALIS*, tuberigen complex, tuberisation

## Abstract

Potato tuber formation is a secondary developmental programme by which cells in the subapical stolon region divide and radially expand to further differentiate into starch‐accumulating parenchyma. Although some details of the molecular pathway that signals tuberisation are known, important gaps in our knowledge persist. Here, the role of a member of the *TERMINAL FLOWER 1*/*CENTRORADIALIS* gene family (termed *StCEN*) in the negative control of tuberisation is demonstrated for what is thought to be the first time. It is shown that reduced expression of *StCEN* accelerates tuber formation whereas transgenic lines overexpressing this gene display delayed tuberisation and reduced tuber yield. Protein–protein interaction studies (yeast two‐hybrid and bimolecular fluorescence complementation) demonstrate that StCEN binds components of the recently described tuberigen activation complex. Using transient transactivation assays, we show that the *StSP6A* tuberisation signal is an activation target of the tuberigen activation complex, and that co‐expression of *StCEN* blocks activation of the *StSP6A* gene by StFD‐Like‐1. Transcriptomic analysis of transgenic lines misexpressing *StCEN* identifies early transcriptional events in tuber formation. These results demonstrate that StCEN suppresses tuberisation by directly antagonising the function of StSP6A in stolons, identifying *StCEN* as a breeding marker to improve tuber initiation and yield through the selection of genotypes with reduced *StCEN* expression.

## Significance Statement

Understanding the mechanism of tuber initiation in potato provides a key route to increasing productivity of a crop that is essential for food security. We demonstrate that a member of the potato phosphatidylethanolamine‐binding protein gene family (*StCEN*) is a key component in the regulation of tuberisation that acts via interaction with the tuberigen activation complex to regulate early tuberisation transcriptional changes.

## INTRODUCTION

Tuberisation in potato is a major photoperiodic developmental programme by which tubers develop from axillary underground buds at the base of the main stem. These buds are activated and grow diagravitropically to form specialised below‐ground branches called stolons. On tuberisation, longitudinal growth of the stolon ceases, whereas the subapical stolon region initiates radial growth (Viola *et al*., 2001). Tuberisation is promoted by long nights, cool temperatures and low rates of nitrogen fertilisation, and depends on the physiological age of the seed tuber (reviewed in Rodriguez‐Falcon *et al*., 2006). The earliness of tuberisation dictates the time to crop maturity and is therefore a crucial factor in potato agronomy. Varieties that reach maturity quickly are particularly beneficial when potato is used as an intercrop or in environments where growing seasons are short due to climatic conditions and disease pressure (Adavi *et al*., 2018).

The molecular mechanisms that control tuber formation have been investigated in detail over the past few decades. Recent breakthroughs have demonstrated that the tuberisation signal is encoded by an ortholog of *FLOWERING LOCUS T* (*FT*) termed *StSP6A* (Abelenda *et al*., 2011; Navarro *et al*., 2011). *StSP6A* and *FT* encode small globular proteins belonging to the phosphatidylethanolamine‐binding protein (PEBP) family. The StSP6A mobile ‘tuberigen’ signal is produced in the leaves and moves to the stolon tip where tuberisation is initiated. Expression of this signal is amplified during transport by an autoregulatory mechanism (Navarro *et al*., 2011) that is not yet fully understood. Genetic screens of various populations and association panels for earliness of tuberisation, using whole‐plant senescence as a proxy for tuber initiation, identified alleles of *CYCLING DOF FACTOR 1* (*StCDF1*) as one of the key components in tuberisation signalling which mediates interaction between the circadian clock and *StSP6A* expression in the leaves (Kloosterman *et al*., 2013). In early maturing *tuberosum* cultivars, naturally occurring alleles of *StCDF1* evade light‐dependent protein destabilisation and enable tuberisation under long days. These findings, together with other results implicating CONSTANS‐like 1 (StCOL1) and Phytochrome B (PhyB) in the day length pathway, led to a detailed model for tuberisation control (Kloosterman *et al*., 2013) in which StCOL1 inhibits tuberisation during long days via the transcriptional control of *StSP5G*, another member of the *FT* gene family that acts as a repressor of *StSP6A* (Abelenda *et al*., 2016). StCOL1 must be stabilised to induce *StSP5G*, in a process that requires active PhyB. As such, the inductive capacity of StCOL1 is predominant when its expression coincides with daytime, which occurs under long days (or short nights). StCDF1 is shown to act as a repressor of StCOL1, while complex formation with the circadian clock components GIGANTEA and FKF1 triggers destabilisation of this negative regulator. In potato genotypes expressing truncated *StCDF1* alleles that lack the C‐terminal FKF1‐interacting region, strict photoperiodic control of tuberisation is then lost due to stabilisation of StCDF1 protein, which leads to constitutive repression of *StCOL1* and defective expression of *StSP5G* (Kloosterman *et al*., 2013).

As well as StSP6A, other phloem mobile elements are important in tuberisation signalling (Hannapel and Banerjee, 2017). These include *StBEL5* mRNA, the transcripts for other *StBEL* family members (Ghate *et al*., 2017) and a number of small regulatory RNAs that affect tuberisation signalling (Martin *et al*., 2009; Eviatar‐Ribak *et al*., 2013; Lehretz *et al*., 2019). StBEL5 functions upstream of StSP6A and is able to induce genes involved in tuber development via its interaction with POTH1, a KNOTTED 1‐type transcription factor (Mahajan *et al*., 2012). Other members of the *BEL1‐like* gene family in potato also encode mobile signals, and StBEL11 and StBEL29 were recently demonstrated to antagonise the function of StBEL5 (Ghate *et al*., 2017). This suggests that, as for flowering, additional pathways may converge to control tuberisation, although it is remarkable that allelic copies encoding truncated forms of *StCDF1* are present in all modern potato cultivars. Indeed, due to their dominant character, these variants had a major role in driving tuberisation under longer day lengths on the introduction of potato to Europe (Gutaker *et al*., 2019), although direct tuber initiation studies showed that genetic variation at loci other than the earliness locus also exerts an effect on tuberisation time (Van den Berg *et al*., 1996; Kittipadukal *et al*., 2012; Zhou *et al*., 2014). Genes underlying these quantitative trait loci (QTLs) remain to be identified.

Arabidopsis FT interacts in the shoot apex with the basic leucine zipper (bZIP) transcription factor FD to form a hexameric complex with 14‐3‐3 proteins, termed the florigen activation complex (FAC). The interaction of these proteins leads to the activation of several downstream MADS‐box floral identity genes such as *APETALA1* and the initiation of floral induction (Abe *et al*., 2005; Wigge *et al*., 2005). The FD protein is also shown to interact with the flowering repressor TERMINAL FLOWER‐1 (TFL1), homologous to CENTRORADIALIS (CEN), to form a transcriptional inhibitory complex repressing the same floral identity genes that are induced by FT (Hanano and Goto, 2011). This indicates that FD is required for TFL‐1 activity, and that FT and TFL‐1 function antagonistically. Lineage‐specific duplications of the *FT‐* and *TFL1‐*like genes have led to redundancy and neo‐functionalisation within species, with far‐reaching impacts on plant architecture. Stable mutations in *TFL1*‐like gene copies have underpinned domestication of crops such as barley, strawberry and legumes (Comadran *et al*., 2012; Iwata *et* *al*., 2012; Kwak *et* *al*., 2012), and in tomato a natural mutation in the *CEN* homolog *SELF‐PRUNING* (Pnueli *et* *al*., 1998) had a major beneficial impact by greatly increasing production in varieties destined for the processing industry.

Recently, an analogous complex termed the tuberigen activation complex (TAC) comprising StSP6A, St14‐3‐3s and StFD‐LIKE1 (StFDL1) has been identified in potato (Teo *et al*., 2016). This complex is thought to have a regulatory role in tuber initiation by acting in an equivalent manner to the FAC. This raises the possibility that potato orthologues of *TFL‐1/CEN* act as tuberisation inhibitors by competing with StSP6A in the tuberigen complex, thus exerting a major regulatory role in tuber initiation and development. We had identified in previous studies a *TFL‐1*/*CEN* orthologue (*StCEN*) on potato chromosome 3 as being the gene responsible for one of the largest effect QTLs for post‐harvest sprout growth (Morris *et al*., 2019). The direct orthologue of StCEN in tomato (Solyc03g026050) has a role in the consistent promotion of vegetative growth and delay of flowering (Hollwey *et al*., 2017).

We demonstrate in this study that in addition to tuber sprouting, StCEN has a major effect on tuber initiation and development, via interaction with the potato TAC StFDL1 and 14‐3‐3 proteins. We propose that competitive effects of StCEN on the function of TAC are part of a central mechanism in the spatial control of tuber formation and suppression of tuber fate identity of tuber sprouts.

## RESULTS

### Expression of *StCEN* in Desiree potato plants

The focus of this study is the functional characterisation of an orthologue of TERMINAL FLOWER 1/CENTRORADIALIS (PGSC0003DMG400014322), a member of the 13‐gene *FT*/*TFL1* family in potato (Abelenda *et al*., 2014), and referred to as *StCEN* in this manuscript. This gene is located on chromosome 3 and was initially identified as being associated with one of the largest effect QTLs for rapid tuber sprout growth (Morris *et al*., 2019).

The expression pattern of *StCEN* in *Solanum* *tuberosum* cv. Desiree plants was determined by quantitative RT‐PCR analyses of leaf, stem, root and tuber samples during 13 weeks of development (Figure S1a in the online Supporting Information). Although transcripts for the *StCEN* gene were detected in all organs, at week 7 its levels were higher in roots than in leaves. However, levels of this transcript increased subsequently in leaves, and by week 9 had reached levels about 2.5‐fold greater than in the stem or the roots. Plants initiated tuber formation around week 6, and levels of *StCEN* transcript in young developing tubers harvested at week 7 were about 2500‐fold lower than in leaves.

Levels of the *StSP6A* transcript, encoding the tuber‐inducing FT signal, were also determined in the same samples (Figure S1b). Notably, *StSP6A* and *StCEN* followed similar patterns of expression in leaves, with the highest expression levels at around week 9. At this developmental stage, *StSP6A* expression was also induced in roots and tubers, but its levels were about 18‐fold and 96‐fold, respectively, lower than those measured in leaves. In stems, *StSP6A* transcripts were highest at week 13, reaching at this age levels similar to those in leaves.

### Characterisation of Desiree *StCEN* transgenic lines

Previously, several independent *StCEN* transgenic lines were generated by *Agrobacterium*‐mediated transformation of *35S‐*promoted overexpression (OE) and RNA interference (RNAi) constructs in potato cv. Desiree (Morris *et al*., 2019). Based on transcript levels in tuber sprouts, two RNAi lines and two OE lines were selected for more detailed phenotypic analysis. Notably, we observed that RNAi lines tuberised much earlier than wild‐type (WT) controls, whereas tuberisation was delayed in OE lines.

**Figure 1:**
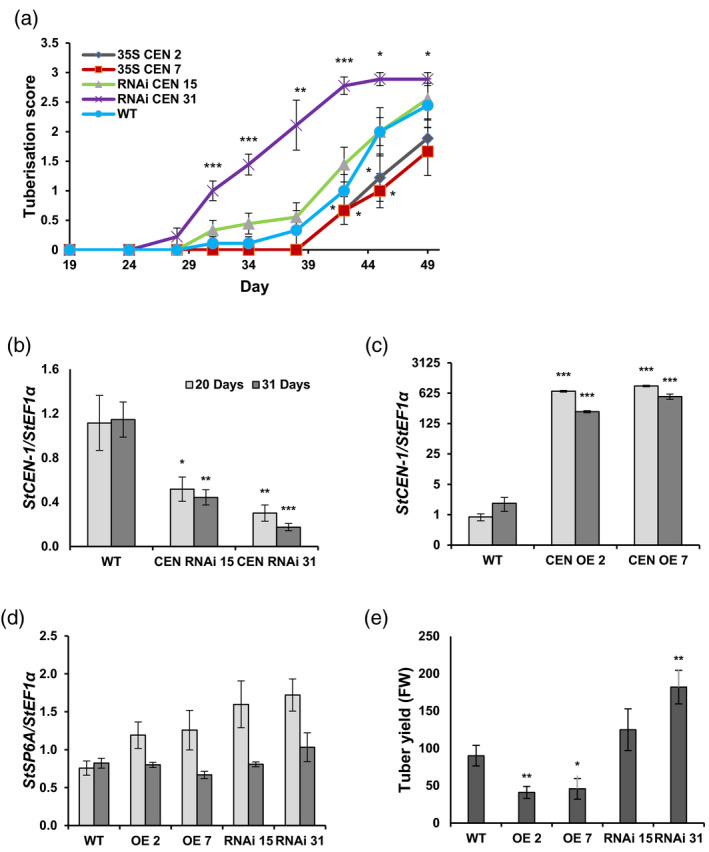
Characterisation of Desiree *StCEN* transgenic lines. (a) Assessment of stolon growth and tuber development in hydroponically grown Desiree *StCEN* transgenic lines compared with wild‐type Desiree control (WT). Stolon/tuber development was scored by visual assessment using the following scale: 0, developing stolons; 1, swelling stolons; 2, developing tubers (<1 cm); 3, tubers >1 cm. All data are represented as mean values ± SE of 12 independent biological replicates. RNAi, RNA interference. (b)–(e) The expression of *StCEN* in Desiree *StCEN* RNAi lines (b), *StCEN* overexpression (OE) lines (c) and *StSP6A* (in RNAi and OE lines) (d) was analysed in leaves from hydroponically grown Desiree plants, harvested at 20 and 31 days after transfer to hydroponic baths under glasshouse conditions. The tuber yield from hydroponically grown plants harvested at 76 days is shown in panel (e) (FW, fresh weight). Expression levels were determined by quantitative RT‐PCR relative to the reference gene *StEF1α*. All data are represented as mean values ± SE of three independent biological replicates and asterisks denote values that were significantly different between transgenic lines and wild‐type controls as determined by Student’s *t*‐test (*P ≤ 0.05; **P ≤ 0.01; ***P ≤ 0.001).

In order to monitor tuberisation time in more detail, plants were grown in a hydroponic system where stolon growth and tuber initiation could be more accurately assessed. For this purpose, plantlets were grown in tissue culture for 4 weeks prior to transfer to hydroponic chambers, in a random block design. Individual plants were visually scored every 3–4 days and a value assigned according to the stage of below‐ground development, using the following scale: 0, developing stolons; 1, swelling stolons; 2, developing tubers (<1 cm); 3, tubers >1 cm (Figure 1a). Both RNAi lines initiated tubers earlier than the WT controls. The RNAi line 31, with the strongest silencing of the endogenous gene, displayed the most rapid stolon formation, tuber initiation and tuber growth. Tuber initiation in these lines was approximately 10 days earlier than in the controls. In contrast, both OE lines exhibited delayed stolon formation and tuber initiation/growth, with the transition to tuberisation taking about four more days than in the controls.

Leaf samples were taken from these plants at days 20 and 31, and the expression levels of *StCEN* and *StSP6A* measured by qRT‐PCR (Figure 1b–d). These gene expression studies confirmed a lower expression of *StCEN* in RNAi lines and elevated expression in OE lines. In contrast, levels of the *StSP6A* transcript were not significantly affected in any of the samples despite the different tuber initiation times (Figure 1d). Tubers were harvested after 76 days and weighed to measure yield (Figure 1e). For both OE lines, tuber yield was significantly less than in the WT controls (by up to about 50%), while RNAi lines showed an increased tuber yield that was particularly significant for RNAi line 31, where it was 102% greater than in the controls.

### Characterisation of the *Solanum andigena* 7540 genotype *StCEN* transgenic lines

Tuberisation in the *S. andigena* 7540 genotype (ADG) is strictly dependent on short day lengths (Navarro *et al*., 2011). To assess the role of *StCEN* in tuberisation more accurately, we produced transgenic *StCEN* RNAi and OE lines in this day‐length‐dependent background. Several independent lines were screened for *StCEN* expression level in the leaf by semi‐quantitative PCR and qRT‐PCR, and three of the strongest RNAi and OE lines were selected for further detailed analysis.

Plants were grown from tissue culture plantlets in controlled environment cabinets under four different day lengths, ranging from 8 to 16 h of light.

**Figure 2:**
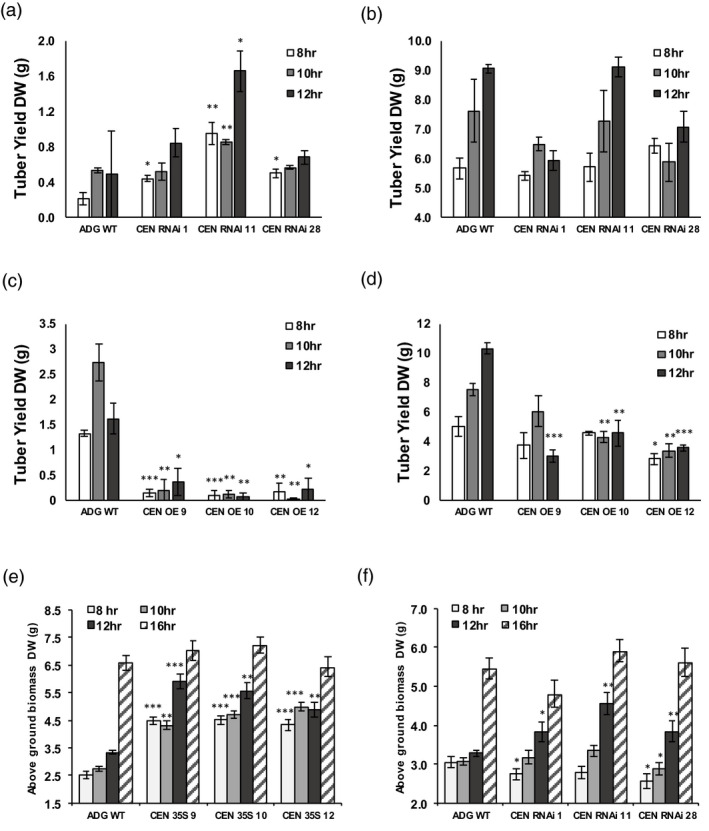
Tuber yield and above‐ground biomass in *StCEN* transgenic *Solanum andigena* 7540 genotype (ADG) lines. Tuber weight yield (DW, dry weight) in ADG RNA interference (RNAi) lines (panels a and b) and ADG OE lines (panels c and d). Plants were grown in pots under growth cabinet conditions at different day lengths (8, 10, 12 h) and were harvested at days 23 and 51 (RNAi lines) and 38 and 60 [overexpression (OE) lines]. All data are represented as mean values ± SE of three independent biological replicates and asterisks denote values that were significantly different between transgenic lines and wild‐type (WT) controls as determined by Student’s *t*‐test (*P ≤ 0.05; **P ≤ 0.01; ***P ≤ 0.001). Above‐ground biomass yield of ADG OE lines (e) and ADG RNAi lines (f). Plants were grown in pots under growth cabinet conditions at different day lengths (8, 10, 12, 16 h) and were harvested at 51 days (RNAi lines) and 60 days (OE lines). All data are represented as mean values ± SE of three independent biological replicates and asterisks denote values that were significantly different between transgenic lines and wild‐type controls as determined by Student’s *t*‐test (*P ≤ 0.05; **P ≤ 0.01; ***P ≤ 0.001).

As for Desiree lines, transgenic ADG RNAi lines initiated tubers earlier and showed more rapid tuber development. Yield data at day 23 (Figure 2a) showed that under 8 h light, tuber yields were significantly higher in *StCEN* RNAi lines than in the WT controls. For plants grown under a 12 h day, tuber yield was also higher by up to about 50% in the RNAi lines, while in these photoperiodic conditions only one of the three WT control plants produced tubers. The higher yield of RNAi lines reflected a trend towards earlier tuberisation under this photoperiod, although this difference was not significant at the *P* > 95% level (Figure S2). Indeed, at later harvest times (51 days; Figure 2b), no differences in tuber yield were observed between the RNAi lines and controls. Also, for 12 h days, tuber yields were higher in both the WT and RNAi plants, indicating that the critical day length for ADG tuberisation is close to this photoperiod. In this regard, none of the plants produced tubers under a day length of 16 h, at either harvest point, consistent with a function of StCEN in suppressing the promotive effects of day length on tuber formation.

In line with this function, tuberisation was significantly delayed in the ADG *StCEN* OE lines compared with the WT, similar to the previous observations in the Desiree background. In OE plants, tubers initiated 15 days later than in the RNAi lines and were harvested shortly after their differentiation in OE lines, on day 38. In this first harvest, yields were significantly lower for these plants compared with the controls, for day lengths of 8, 10 and 12 h (Figure 2c). A reduced tuber yield of the OE plants was also observed after 60 days (Figure 2d). In this second harvest, tuber yield of controls in plants grown under 12 h light was also higher than for plants grown under 8 and 10 h days, and a significant reduction in tuber yield was observed in these conditions in all three OE lines (Figure S2). However, in plants grown under 8 h days, only the stronger OE line showed a statistically significant lower yield than the WT.

Above‐ground biomass was, on the other hand, significantly increased in all OE lines compared with the WT controls, independently of whether they were grown under day lengths of 8, 10 or 12 h (Figure 2e). Also, whereas RNAi lines had a reduced above‐ground biomass compared with WT when grown under 8 h days (Figure 2f), no significant difference in above‐ground biomass was observed for either OE or RNAi transgenic lines under 16 h days, when tuberisation was inhibited in all genotypes (Figure 2e,f). In RNAi lines grown under 10 or 12 h days, there was a variable impact on above‐ground biomass, with an increase observed for all three lines grown under 12 h days, despite there being no significant impact on tuber yield. Overall, these findings indicate that the increase in above‐ground biomass in OE lines relies on a reduction or total inhibition of tuberisation, and that accelerated tuber initiation and development in the RNAi lines has a less clear‐cut impact on above‐ground biomass.

**Figure 3:**
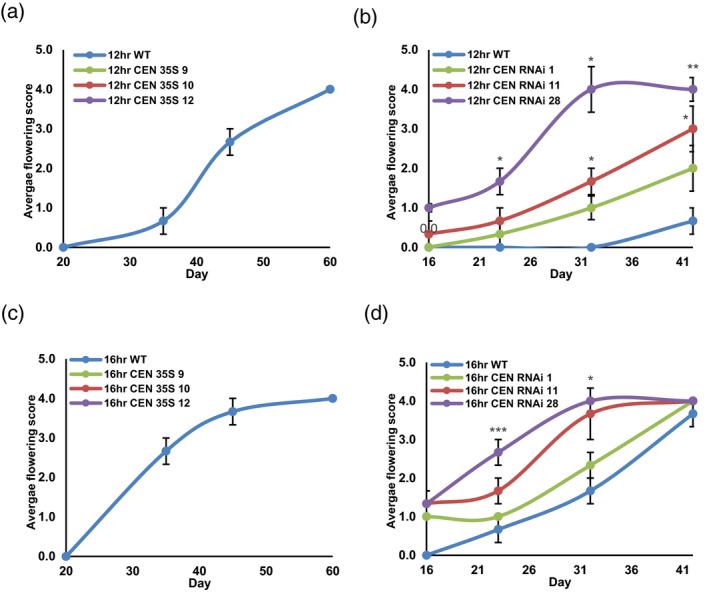
Floral development of *Solanum andigena* 7540 genotype (ADG) transgenic lines. Floral development in ADG *StCEN* overexpression (OE) (panels a and c) and RNA interference (RNAi) (panels b and d) lines grown in cabinets under day lengths of 12 h (a,b) and 16 h (c,d). All data are represented as mean values ± SE of six independent biological replicates. Asterisks denote values that were significantly different between transgenic lines and wild‐type (WT) controls as determined by Student’s *t*‐test (*P ≤ 0.05; **P ≤ 0.01; ***P ≤ 0.001).

Major effects on flower development were also observed in the *StCEN* transgenic lines. For WT, RNAi and OE lines, no flower buds or open flowers were observed in plants grown under 8 and 10 h days. In addition, no signs of flower bud initiation were observed in OE lines grown under 12 and 16 h days (Figure 3a,c). In the RNAi lines, however, flower buds developed up to 15 days earlier than the WT under 12 h light, and 7 days earlier under 16 h days (Figure 3b,d). Also, open flowers were observed earlier in both the WT and RNAi lines under 16 h compared with 12 h days.

**Figure 4:**
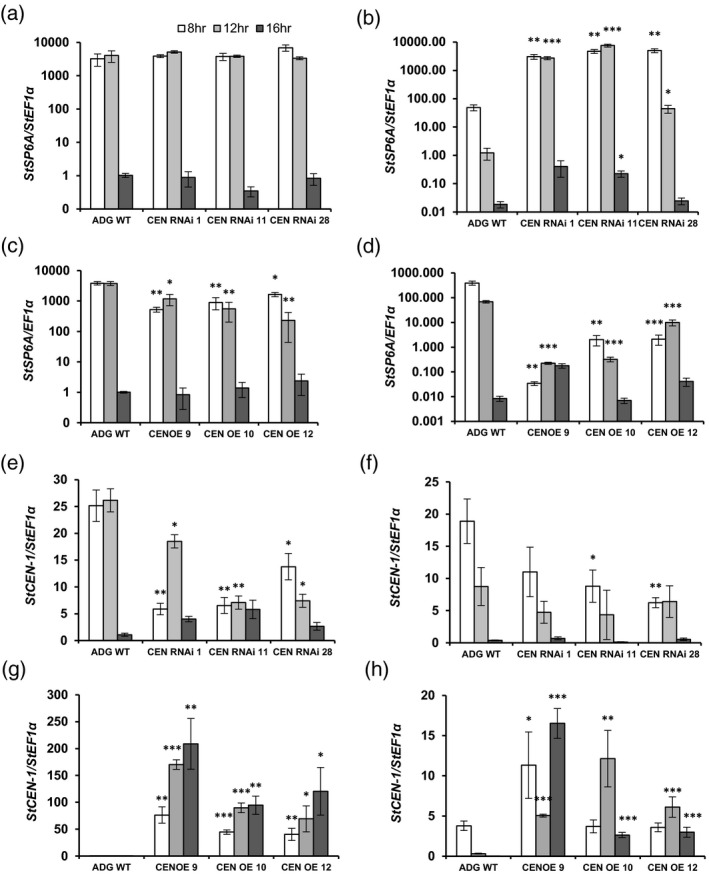
*StSP6A* and *StCEN* expression patterns in *Solanum andigena* 7540 genotype (ADG) transgenic lines. The expression of *StSP6A* (a–d) and *StCEN* (e–h) in leaves (a,c,e,g) and stolons (b,d,f,h) of ADG RNA interference (RNAi) lines (a,b,e,f) and overexpression (OE) lines (c,d,g,h) was analysed in plants cultivated in growth cabinets for 23 days (RNAi lines) and 38 days (OE lines) under day lengths of 8, 12 or 16 h. Expression levels were determined by quantitative RT‐PCR relative to the reference gene *StEF1α* and are expressed on a log scale. All data are represented as mean values ± SE of three independent biological replicates and asterisks denote values that were significantly different between transgenic lines and wild‐type (WT) controls as determined by Student’s *t*‐test (*P ≤ 0.05; **P ≤ 0.01; ***P ≤ 0.001).

We analysed the expression levels of *StSP6A* and *StCEN* in the 8, 12 and 16 h day leaf and stolon samples from RNAi (day 23) and OE (day 38) lines (Figure 4a–d). Notably, differences in the *StSP6A* expression pattern were more marked in stolons than the leaf samples, and these effects were opposite in the RNAi (Figure 4a,b) and OE lines (Figure 4c,d). Differences in *StSP6A* expression were minimal in leaves. However, in stolon samples from all three RNAi lines, the *StSP6A* expression level was markedly higher (by more than 100‐fold) than in the WT under all day lengths (Figure 4b). A similar trend was observed in OE lines (Figure 4c,d). Whereas *StSP6A* expression was reduced by only up to fourfold in OE leaves, in stolon samples from OE plants grown under 8 and 12 h days, *StSP6A* transcript levels were reduced by 11 500‐fold (Figure 4c,d). This trend was not observed in stolons from 16 h day plants, where *StSP6A* approached the limits of detection in both the WT and transgenic lines.

*StCEN* expression was also measured in leaf and stolons from the transgenic lines and WT controls (Figure 4g,h). Significantly higher levels of *StCEN* expression (by up to 200‐fold) were detected in the leaves of OE lines (Figure 4g). In stolons, *StCEN* expression levels were also higher compared with WT values, although the fold increase was less than in leaves (a maximum of 16‐fold; Figure 4h). *StCEN* expression showed the opposite pattern in *StCEN* RNAi lines (significantly lower in leaf samples from *StCEN* RNAi lines than in the corresponding controls; Figure 4e), while differences in stolons were statistically significant only for 8 h day samples from RNAi lines 11 and 28 (Figure 4f).

### Transcriptional profiles associated with altered *StCEN* expression in stolons from transgenic lines

Transcriptomic analyses were performed using a custom Agilent microarray containing probes representing transcripts of all 39 031 protein‐coding genes predicted from the potato genome sequence (Potato Genome Sequencing Consortium, 2011). We compared transcriptional profiles in non‐induced stolons from the ADG OE, RNAi and WT lines.

A pairwise comparison of transcripts from RNAi lines and WT identified 4635 genes that were differentially expressed (log_2_ ≥ 0.5 or ≤0.5, *P* ≤ 0.05). Using *k*‐means clustering this gene set was grouped into four main clusters (Figure S3). Cluster 1 (1215 genes) contained transcripts weakly upregulated in RNAi lines whereas Cluster 2 contained 1260 transcripts strongly upregulated in RNAi lines compared with WT and OE lines. Clusters 3 (1202 genes) and 4 (958 genes) contained transcripts weakly (Cluster 3) or strongly (Cluster 4) downregulated compared with the WT and OE lines. Lists of genes associated with the four *k*‐means clusters are provided in Table S1.

**Figure 5:**
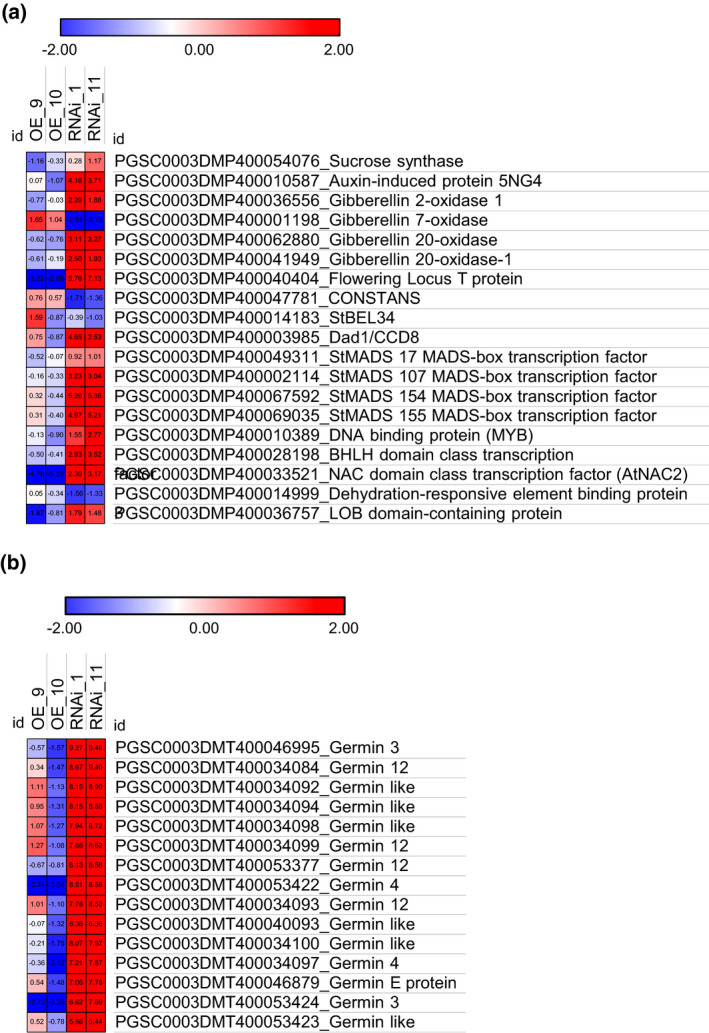
Heat map representation of selected transcripts associated with early tuberisation in stolons from *Solanum andigena* 7540 genotype (ADG) transgenic lines. Heat map representation of selected transcripts associated with early tuberisation (a) and germin transcripts (b) which are differentially expressed in stolons from ADG *StCEN* RNA interference (RNAi) lines compared with wild‐type (WT) and overexpression (OE) lines. Heat maps were constructed using Morpheus matrix visualisation and analysis software, (https://software.broadinstitute.org/morpheus) and log_2_‐transformed normalised expression values.

Based on previous literature, we selected genes that had been associated with early tuberisation and show that these genes are in fact upregulated in the RNAi lines compared with WT and OE lines (Figure 5a). Further analysis revealed that three MADS‐box genes encoding StMADS17 (PGSC0003DMG400028359), StMADS107 (PGSC0003DMG400001143) and a previously undescribed StMADS box factor (PGSC0003DMG400046931) located on chromosome 10 are differentially expressed in RNAi stolons. An earlier genome‐wide survey of the potato MADS‐box family (Gao *et al*., 2018) identified *StMADS1*, ‐*3*, ‐*11*, ‐*12*, ‐*13*, ‐*17* and ‐*27* as potential downstream targets of StSP6A. In our analysis, however, only *StMADS17* showed a strong and consistent upregulation in stolons of the StCEN RNAi lines (Figure 5), while the rest of the MADS‐box genes potentially targeted by StSP6A did not show any altered expression (Data S2). More remarkably, in the set of strongly upregulated genes in Cluster 2, we identified several transcripts encoding for germin‐like proteins, described in Arabidopsis as located in the plasmodesmata (Ham *et al*., 2012). Transcripts annotated as *germin3*, ‐*4* and ‐*12* are in fact expressed at levels between about 200‐ and 700‐fold higher in the stolons from RNAi lines than in those from controls (Figure 5b), suggesting that this family of proteins may have a relevant role in the switch in from apoplastic to symplastic sucrose unloading, marking initiation of tubers (Viola *et al*., 2001).

### StCEN interacts with components of the tuberigen complex

**Figure 6:**
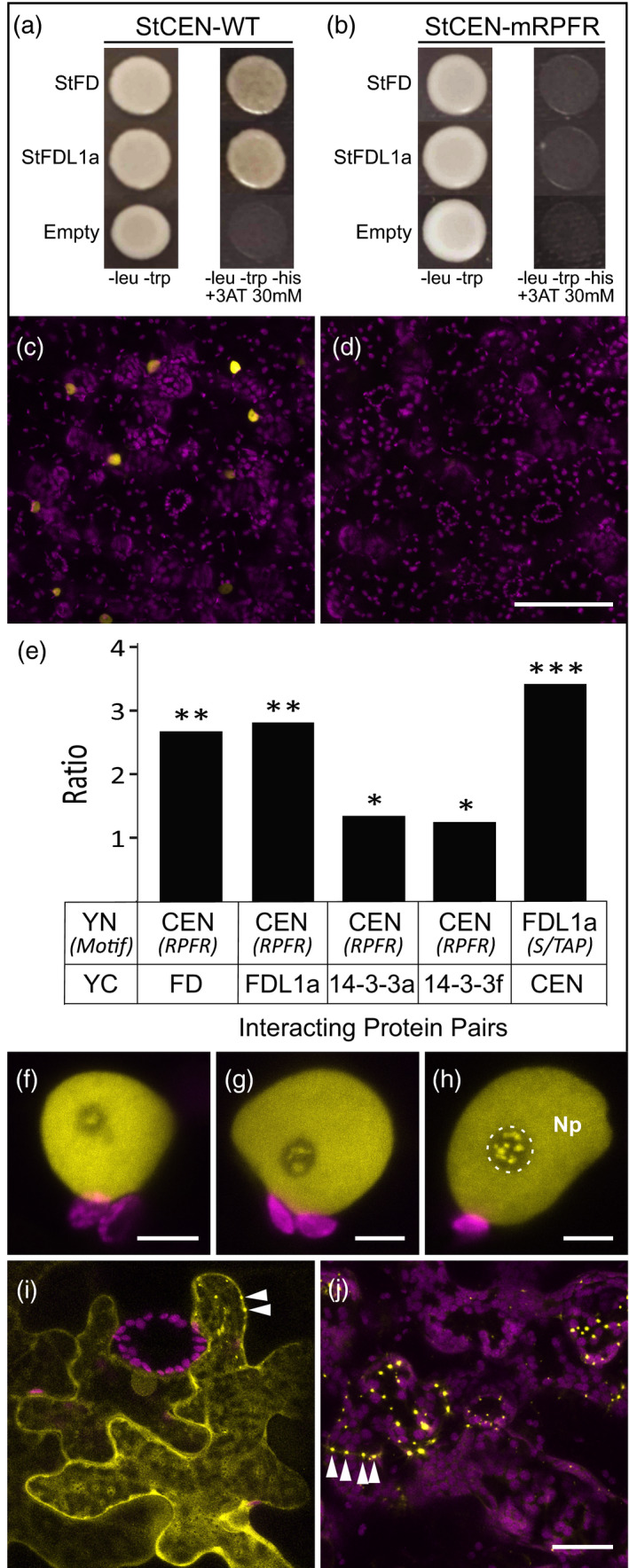
Interaction between StCEN and StFD and StFDL1a proteins. Yeast two‐hybrid assays demonstrating the interaction between StCEN and StFD and StFDL1a proteins (a) and loss of interaction in the StCEN‐mRPFR mutant (b) (3AT, 3‐amino‐1,2,4‐triazole). The empty vector pDEST22 was used as a negative control. Fluorescence from reconstituted yellow fluorescent protein (YFP) is shown in yellow and chlorophyll fluorescence (chloroplasts) is shown in magenta. (c), (d) Low‐magnification *z*‐stack projections of tissue infiltrated with paired constructs: YN‐StCEN and YC‐FD (c) or YN‐StCENmRFPR and YC‐FD (d), collected from opposite half‐leaves under the same imaging conditions. Fluorescence was primarily in nuclei, with a very weak cytoplasmic signal occasionally visible. The wild‐type protein fusion (c) showed brighter nuclear fluorescence than the mutant protein fusions (d). Quantification of the difference in interaction between wild‐type and mutant protein pairs is shown in (e). Each bar represents the ratio of mean quantified fluorescence in bimolecular fluorescence complementation experiments between one YC partner (the C‐terminal portion of split‐YFP) and a pair of YN proteins (N‐terminal portion of split‐YFP); wild‐type versus mutant in each case (i.e. the left‐hand bar shows the ratio of fluorescence between (YC‐FD+YN‐StCEN) and (YC‐FD+YN‐StCENm^RFPR^). Mean pixel intensity values in the YFP channel of random fields from maximum intensity projections (*n* ≥ 10) were used to calculate the ratios, where each pair of constructs were imaged under the same conditions from opposing half‐leaves. The larger the ratio, the greater the reduction in fluorescence observed with the mutant protein compared with the wild‐type. The significance of the reduction in fluorescence was tested using a one‐tailed *t*‐test and represented using asterisks: *P ˂ 5 × 10^3^; **P ˂ 1 × 10^6^; ***P ˂ 1 × 10^7^. (f)–(h) Higher‐magnification images of representative nuclei showing the localisation of reconstituted YFP in the following pairs of constructs: YN‐StCEN and YC‐FD (f); YN‐StCEN and YC‐FDL1a (g); YN‐StCENm^RFPR^ and YC‐FD (h). In each case, the protein interaction leads to YFP fluorescence in the nucleoplasm and nucleolus. Within the nucleolus (outlined in (h) with a dotted line), fluorescence is concentrated in discrete bright bodies. The nuclear localisation is consistent for both wild‐type and mutant protein combinations. (i), (j) Showing the fluorescence when YN‐StCEN and YC‐14‐3‐3 are co‐expressed. In epidermal tissue (i), fluorescence is predominantly cytoplasmic, with some cells also showing bright punctate spots at the cell periphery (arrowheads). In contrast, mesophyll cells (j) typically show no, or low levels, of cytoplasmic signal, and fluorescence is highly localised to bright, punctate spots which again localise to the cell periphery, evident at cell junctions (arrowheads). The scale bar in (d) represents 100 µm (for c and d), 5 µm in (f)–(h) and in (j) 20 µm (for i and j).

The phenotype of the *StCEN* transgenic lines, in both the Desiree and ADG backgrounds, suggests that StCEN is a negative regulator of tuberisation. The TFL‐1 CEN homologue has been shown in Arabidopsis to function as a floral inhibitor that competes with FT for interaction with the FD bZIP factor in the assembly of the florigen activation complex. Binding of TFL‐1 to the 14‐3‐3s and FD proteins does not activate downstream genes and results in inhibition of flowering (Hanano and Goto, 2011). We therefore investigated whether the inhibitory effects of StCEN on tuberisation could be due to competition with StSP6A for interaction with the FD‐like factors comprising the tuberigen activator or TAC complex (Teo *et al*., 2016). Firstly, we amplified the open reading frames (ORFs) of recently identified potato FD (StFD) and FD‐like (StFDL1a) proteins from a Desiree cDNA and used these fragments to build yeast two‐hybrid (Y2H) constructs to test whether StCEN is able to bind each of the putative tuberigen components. Based on these Y2H assays, StCEN was observed to physically interact with StFD and StFDL1a (Figure 6a). Also, the StCEN protein shares four amino acid residues that are conserved in other FT family members and are essential for binding 14‐3‐3 proteins (Teo *et al*., 2016). Thus, a mutated *StCEN* construct in which these four amino acid codons were replaced (R64K/P96L/F103A/R132K) was used to test whether interaction with StFD and StFDL1a was dependent on 14‐3‐3 binding as observed for StSP6A (Teo *et al*., 2016). For the StCENm^RPFR^ mutant, no interaction with StFD or StFDL1a was detected in the Y2H interaction assays (Figure 6b), demonstrating that this interaction is mediated by 14‐3‐3s.

Bimolecular fluorescence complementation (BiFC) with split‐yellow fluorescent protein (YFP) was used to confirm and localise these interactions *in planta*. Since the two halves of YFP were reported to have a propensity to reassemble in the absence of a *bona fide* protein–protein interaction, resulting in a weak fluorescent false‐positive signal (Horstman *et al*., 2014), a quantitative approach was used for these studies. Differences in signal strength were measured after co‐infiltration with the two split‐YFP protein fusions, using negative controls where the protein binding motif had been mutated in one of the interacting partners. In this way, mean pixel intensities for yellow fluorescence were determined from low‐magnification images collected from random fields on the two opposing agro‐infiltrated half‐leaves. Each half‐leaf expressed one putative interacting protein fused to the C‐terminal portion of split‐YFP (YC) in combination with either WT or mutant forms of the other putative interacting protein fused to the N‐terminal portion of split‐YFP (YN).

When YN‐StCEN was co‐expressed with YC‐FD, yellow fluorescence was observed primarily in the nuclei of cells, clearly visible at low magnification as a single, bright spot in each fluorescent epidermal cell (Figure 6c). In contrast, a much weaker yellow fluorescence was observed in cells that co‐expressed YN‐StCENm^RPFR^ with YC‐FD and imaged using the same microscope settings (Figure 6d). Similar fluorescence patterns were obtained for interactions of StFDL1a with StCEN and StCENm^RPFR^.

Quantitative imaging of random fields similar to those shown in Figure 6(c,d) was used to confirm that the YN‐StCEN fusion gave significantly higher levels of yellow fluorescence than the YN‐StCENm^RPFR^ fusion in combination with the YC‐StFD and YC‐StFDL1a fusions (Figure 6E, Data S3).

Higher‐magnification images of single nuclei showed that reconstituted YFP fluorescence was distributed throughout the nucleoplasm and in discrete subnucleolar bodies (Figure 6f–h). The same pattern of fluorescence was seen for both WT and mutant forms of YN‐StCEN, when expressed in combination with either YC‐StFD or YC‐StFDL1a, although the signal obtained for StCENm^RPFR^ was much weaker than that for the WT protein.

StSP6A was described to interact with a strong binding affinity with each of the 11 14‐3‐3 proteins identified in potato, and this interaction is required for binding of StSP6A to FD (Teo *et al*., 2016). Thus, it was suggested that the apparent StSP6A and StFD/StFDL1a interaction seen in the Y2H system was probably mediated by the endogenous yeast 14‐3‐3 proteins (Teo *et al*., 2016). We therefore assessed the interaction of StCEN with two selected potato 14‐3‐3 proteins (St14‐3‐3a or St14‐3‐3f), both in Y2H and plant cell BiFC, to investigate whether StCEN binds these proteins. Interaction of these two proteins in Y2H was weak, but a clear fluorescent signal could be detected in BiFC assays. Quantification of the yellow fluorescence obtained with YN‐StCEN fusions showed that this was significantly greater than for the YN‐StCENm^RFPR^ fusions in plant cells infiltrated with either the YC‐St14‐3‐3a or the YC‐St14‐3‐3f fusions (Figure 6e).

In epidermal cells infiltrated with the YN‐StCEN and YN‐StCENm^RFPR^ proteins, in combination with YC‐St14‐3‐3a and YC‐St14‐3‐3f, yellow fluorescence was primarily observed in the cytoplasm (Figure 6i). However, a subpopulation of epidermal cells expressing the WT YN‐StCEN and YC‐St14‐3‐3a fusions also showed punctate fluorescent spots at the cell periphery (Figure 6i, arrows). In contrast, the fluorescent signal of mesophyll cells was fainter in the cytoplasm (Figure 6j), while it displayed a punctate distribution in the cell periphery, which was observed as aligned spots at the junction between two cells (Figure 6j, arrows).

The StFD and StFDL1a proteins share a conserved C‐terminal S/TAP motif, whose phosphorylation is essential for binding 14‐3‐3 proteins and for their regulatory function (Teo *et al*., 2016). A T224A substitution in this motif was shown to disrupt interaction of potato StFDL1a with both StSP6A and St14‐3‐3 proteins (Teo *et al*., 2016). The T224A mutation was introduced into YN‐StFDL1a, both WT and mutant forms of this protein giving rise, on co‐expression with YC‐StCEN, to YFP fluorescence that was primarily localised in the nucleus (cf. Figures 6f–h). However, quantification of the YFP signals showed that the T224A mutant protein leads to a significantly weaker signal than the WT (Figure 6e), in a similar way to that observed for mutations in StCEN disrupting the 14‐3‐3 interaction motif, in support of interaction with 14‐3‐3s being essential for TAC complex formation.

### StCEN suppresses TAC activation of the *StSP6A* and *germin3* genes

**Figure 7:**
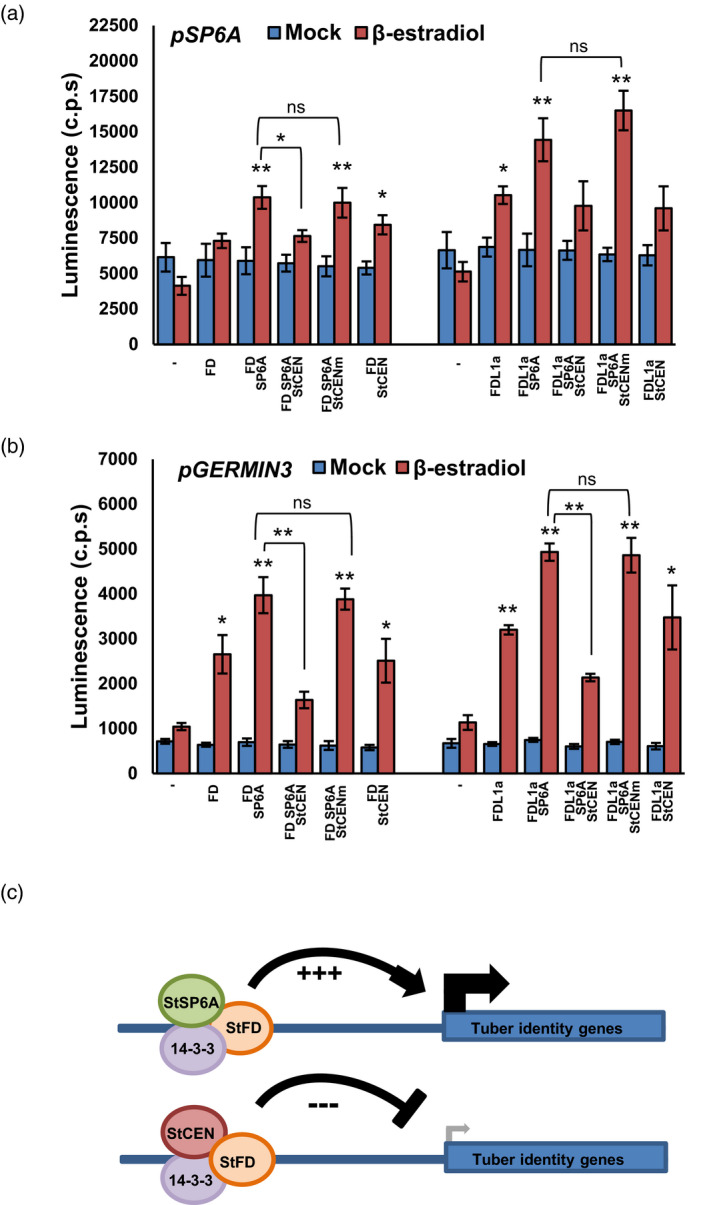
Transactivation of the *StSP6A* and *Germin3* promoters and model for action of the tuberigen activation complex (TAC). (a), (b) Luciferase (LUC) activity luminescence (counts per second, c.p.s.) from transient expression studies of the effector constructs expressing the StFDL1a, StCEN and StSP6A proteins under control of the XVE estradiol‐inducible (FDL1) and CaMV *35S* constitutive (StSP6A, StCEN) promoters co‐transformed with the 2.64 kb *StSP6A* promoter fused to the LUC reporter gene (*p6A*::LUC) (a) and a 1.84 kb *StGermin3* promoter fused to the LUC reporter gene (*pgermin3*::LUC) (b). *Agrobacterium tumefaciens* cells containing each of these constructs were co‐infiltrated into *Nicotiana benthamiana* leaves and luciferase activity was measured at intervals of 1 h for 16 h. Error bars represent the SEM of *n* = 12 leaf discs at the 10 h time point. Studies were repeated three times with similar results. Values that were significantly different were determined by Student’s *t*‐test (*P ≤ 0.05; **P ≤ 0.01). (c) Proposed model for TAC regulation; the StSP6A, StFDL1a and 14‐3‐3s tuberigen activation complex activates transcription of tuber identity genes. The complex containing StCEN, StFDL1a and 14‐3‐3 proteins suppresses transcription of tuber identity genes. StCEN competes with StSP6A for the formation of an active TAC complex.

To demonstrate that StCEN and StSP6A exert antagonistic effects on *StSP6A* transcription, transient transactivation assays were performed. Our observation that alterations in *StSP6A* expression levels were higher in the stolons of *StCEN* OE and RNAi plants than in the leaves indeed suggested that *StSP6A* is a direct target of the StFDL1a factor in the stolons. To test this hypothesis, a 2.64 kb promoter region upstream of the *StSP6A* start codon was fused to the luciferase gene (*p6A*::LUC) and used as a reporter construct in transactivation assays. This reporter was agro‐infiltrated into *Nicotiana benthamiana* leaves, alone or in combination with effector constructs expressing the StFDL1a, StCEN and StSP6A proteins under control of the *XVE* (StFDL1a) or *35S* promoters (StSP6A, StCEN and StCENm), to the estradiol‐responsive or constitutive expression of these proteins (Figure 7a). Leaves co‐expressing the StSP6A and StFDL1a proteins showed in these assays near to three‐fold higher LUC expression levels than those infiltrated with the *p6A*::LUC reporter alone. Higher LUC levels were also observed on single expression of the StFDL1a factor, although fold induction in this case was less than with the combined StSP6A and StFDL1a proteins. Increased LUC activity was also only detected after estradiol application, demonstrating that StFDL1a is required for this activation. StSP6A‐dependent induction of its own promoter, on the other hand, was suppressed by StCEN but not the StCENm mutant protein defective in 14‐3‐3 interaction (Figure 7a). These results suggest that the co‐expressed StSP6A and StFDL1a proteins interact with *N*. *benthamiana* 14‐3‐3s in the formation of a TAC targeting the p*6A* promoter, while StCEN suppresses this activation, probably by interfering with recruitment of StSP6A into this complex. Moreover, our observation that WT StCEN, but not StCENm defective in 14‐3‐3 interaction, suppresses StSP6A co‐activation of the LUC reporter, indicates that 14‐3‐3s have a critical role in facilitating interaction of StSP6A with StFDL1a, and therefore in TAC formation.

The greatly enhanced expression of *germin* transcripts in StCEN RNAi lines compared with controls prompted us to investigate whether these genes are also targeted by the TAC. We selected *germin3* (PGSC0003DMT400046995) for these analyses as it was the most strongly upregulated gene copy and has a predicted transit peptide. A construct containing 1845 nucleotides upstream of the presumed start methionine codon was fused to luciferase and used as a reporter as described for the *StSP6A* reporter. As for the *StSP6A* reporter, co‐infiltration with the StFDL1a and StSP6A constructs resulted in a strong induction of the *germin3* reporter (Figure 7b). Co‐expression of StCEN with StFDL1a and StSP6A significantly reduced activation of the *germin3* reporter, whereas StCENm mutated in the amino acids required for 14‐3‐3 protein interaction lacked any inhibitory effect. Overall, these data strongly indicate that *germin3* is a direct target of the TAC complex.

## DISCUSSION

### The role of *StCEN* in the tuber life cycle

Previously we demonstrated a powerful effect of changes in *StCEN* expression on the growth of tuber sprouts during storage (Morris *et al*., 2019). Decreased *StCEN* expression levels in RNAi lines were associated with higher rates of tuber sprout growth, whereas *StCEN* OE transgenic lines exhibited a lower rate of sprouting than controls. In this study, we extended these observations by showing that *StCEN* also has a role in tuber initiation and development. We tracked *StCEN* transgenic lines in the variety Desiree for stolon formation, tuber initiation time and tuber growth using hydroponically grown plants, which facilitated observation of stolon and tuber development without disrupting plant growth. In this system, *StCEN* RNAi lines tuberised significantly earlier (as much as 10 days in some transgenic lines) than controls, while *StCEN* OE lines displayed reduced rates of stolon growth and delayed tuber initiation (Figure 1). Similar effects on tuber initiation and development were also observed in transgenic lines in the ADG background. Interestingly, in this strict day length‐dependent genotype, effects of *StCEN* silencing in promoting tuberisation were more prominent under short days (Figure 2a), whereas the tuberisation delay in OE lines was stronger under long days (Figure 2d). In the ADG genotype, expression of the *StSP6A* tuberisation signal is induced in short days. Day length‐dependent effects of *StCEN* mis‐expression therefore indicate that StCEN inhibits tuber initiation and tuber development in an *StSP6A‐*dependent fashion.

Associated with the delayed tuberisation in ADG OE lines there was an increase in above‐ground biomass, which suggests that carbon partitioning to tubers is reduced in these plants. This effect was less marked in the RNAi lines, although there was a trend towards a stronger reduction of above‐ground biomass for short days, where effects of promotion of tuber initiation were also most obvious. We also noted significant effects of StCEN on flower development, with delayed flowering in OE plants and the opposite for RNAi lines. Interestingly, the recent study of Plantenga *et al*. (2018) demonstrated that flower bud development is improved on reducing *StSP6A* expression and impaired when *StSP6A* expression increases. Our observations were consistent with this model, with enhanced rates of flower development observed in WT ADG plants under long days, when StSP6A transcript levels are not induced. However, in the ADG OE lines, *StSP6A* transcript levels were reduced in leaves compared with the WT, and the rate of floral development was also decreased. Thus, interaction between FT and FT‐like signals seems to be complex and requires further studies to be fully understood. Actually, given the antagonistic effects of StSP6A and StCEN, it is rather difficult to understand why the expression patterns of these genes closely match (Figure S1), although the expression patterns are not yet known at high resolution. Nevertheless, a similar scenario is seen in Arabidopsis where *TFL1* is activated by increased expression of *FT*, despite the antagonistic roles of TFL1 and FT in controlling flowering (Jaeger *et al*., 2013). A model has been proposed for floral transition, where upregulation of *TFL1* in direct correlation with *FT* is necessary to obtain distinct developmental fate outcomes for different cells. Indeed, the concept that the balance between FT and TFL1 has a major role in controlling how apical meristem cells give rise to different structures is gaining traction (Plantenga *et al*., 2018; Moraes *et al*., 2019).

### Interaction of StCEN with components of the tuberigen complex

The TAC comprises StSP6A, St14‐3‐3s and StFDL1a and plays a major role in promoting tuber initiation by acting in an analogous manner to the well‐characterised florigen‐activation complex (Teo *et al*., 2016). TFL1 (analogous to CEN) is shown to compete with FT for FD interaction, leading to a complex that does not activate flowering. An antagonistic function to FT has indeed been demonstrated for TFL1 homologs of Arabidopsis (Hanano and Goto, 2011), rice (Kaneko‐Suzuki *et al*., 2018), maize (Danilevskaya *et al*., 2010) and kiwi fruit (Varkonyi‐Gasic *et al.*, 2019). Thus, we hypothesise that the negative effects of StCEN on tuber initiation and development could be explained by a competitive activity with TAC function, in a manner related to that reported for the FAC complex. Our Y2H and BiFC experiments clearly demonstrate that StCEN interacts with StFD and StFDL1a. The amino acid residues that are required to facilitate interaction of PEBP with 14‐3‐3 proteins and subsequent FAC assembly are conserved in StCEN. Mutation of these amino acids in the potato StCEN protein resulted in a weaker interaction with endogenous *N*.* benthamiana* 14‐3‐3s and the co‐expressed FD/FDL1 proteins, consistent with 14‐3‐3s having a central role in TAC assembly (Figure 6). Furthermore, mutation of the FD S/TAP motif prevents StCEN and FD interaction (Figure 6e), which further supports our hypothesis that negative effects of StCEN on tuber initiation and development are mediated by competition with StSP6A for binding to the FD/FDL1 proteins, which impairs formation of a transcriptionally active TAC. The RNAi line 31, with the strongest silencing of the endogenous gene, displayed the most rapid stolon formation, tuber initiation and tuber growth (Figure 1). However, the relationship between transcript level and phenotype depends on translation to protein and then competitive interaction with SP6A in the tuberigen complex, so a direct level of proportionality might not be expected.

### Impact of StCEN on StSP6A gene expression

To analyse the effects of StCEN on expression of *StSP6A*, we measured *StSP6A* transcript levels in leaves and stolons of *StCEN* lines (Figure 4). Notably, a reduction in *StCEN* expression did not significantly alter *StSP6A* transcript levels in the leaves, both in Desiree and ADG *StCEN*‐RNAi lines, despite the earlier tuber initiation and developmental phenotype of these plants. In contrast, levels of the *StSP6A* transcript were induced by over 100‐fold in the stolons of ADG RNAi lines with respect to the WT controls. Strong *StSP6A* gene activation was actually observed independent of day length conditions in the stolons of the three tested lines, suggesting that StCEN has a major role in suppressing *StSP6A* expression in these organs. Consistent with a stolon‐specific function of the StCEN protein, no significant effects on *StSP6A* expression levels in the leaves were observed for the hydroponically grown OE Desiree plants (Figure 1). Likewise, suppression in *StSP6A* expression levels in the ADG *StCEN* OE lines was much greater in stolons than in leaves.

Expression of the tuber‐inducing *StSP6A* signal was shown to be amplified in the stolon by an autoregulatory mechanism (Abelenda *et al*., 2014), although the molecular details, timing and localisation of this process are unknown. It is also known that the *StSP6A* signal must be expressed during tuber development to maintain tuber growth (Hancock *et al*., 2014). Based on the observed effects of altered *StCEN* expression on *StSP6A* activation in the stolons and the interaction of StCEN with the TAC components, we propose that the *StSP6A* gene is a downstream activation target of the TAC complex. Further evidence for this transcriptional control is provided by the transient transactivation studies in *N*. *benthamiana* leaves, where StCEN and StSP6A were found to, respectively, suppress and enhance StFDL1a‐dependent activation of the *p6A*::LUC reporter. Similar regulation was observed for StFD, although the effects were weaker than with the bZIP StFDL1a factor (Figure 7). These findings lead us to propose that the TAC is involved in *StSP6A* transcription in the stolons, in addition to directly regulating the expression of tuber identity genes (Figure 7b). As expected from the early tuberisation phenotype of the CEN RNAi lines, our transcriptional profiling analyses demonstrated an enhanced expression of StSP6A as well as many tuber‐expressed genes, including those encoding patatin, sucrose synthase and gibberellin 20‐oxidases in the stolons. Also, although their function in tuber transition remains to be characterised in detail, several MADS‐box genes were identified as showing enhanced expression in the RNAi lines (MADS 17 107 154; Figure 5) and therefore potentially constitute additional TAC targets. Of these, *StMADS17* was previously described to be strongly upregulated in stolons (Gao *et al*., 2018) and shown to upregulated in the strongly induced to tuberise transgenic SES lines described by Lehretz *et al*. (2019). StMADS17 has homology to SEPALLATA 4 (SEP 4) from Arabidopsis, with a reported role in the regulation of floral meristem development (Ditta *et al*., 2004).

Several members of the germin gene family were also identified as showing a strongly upregulated expression in StCEN RNAi stolons compared with controls (Figure 5b). Our transactivation experiments provide compelling evidence that *germin3*, the most strongly upregulated of these transcripts, is a direct target of the TAC. Germins and germin‐like proteins (GLPs) are evolutionarily conserved ubiquitous plant glycoproteins belonging to the cupin superfamily (reviewed in Barman and Banerjee, 2015), to which many different functions have been ascribed. As well as having enzymatic activities including oxalate oxidase, superoxide dismutase, ADP glucose pyrophosphatase/phosphodiesterase and polyphenol oxidase, GLPs are thought to promote protection against several biotic and abiotic stresses. Some evidence connects GLPs to plasmodesmal function, as two pumpkin GLPs (PDGLP1 and PDGLP2) were proposed to modulate phloem‐mediated resources allocation between the primary and lateral root meristems (Ham *et al*., 2012). In potato, a major factor contributing to tuber induction and dormancy break is symplastic connectivity, which controls the flux of carbohydrates to meristematic tissues (Hancock *et al*., 2008). Symplastic connectivity between the phloem and the tuber apical meristem is controlled by the presence or lack of functional plasmodesmata at key cell and tissue interfaces. The potential link between germins and plasmodesmal function is therefore intriguing and could connect tuberisation signalling with symplastic connectivity, a hypothesis that will be tested in future work.

In this work, we demonstrate that the *StCEN*/*TFL1* homolog is a major determinant in the control of the potato tuber life cycle, due to inhibitory activity of the TAC complex. In addition to its initially reported impact on tuber sprout growth rate (Morris *et al*., 2019), here we show that StCEN has a pivotal role in modulating the timing of tuber initiation and the rate of tuber development. From a commercial perspective, acceleration of tuber formation is an important trait, of particular interest in regions with a short growing season. Although allelic variation in *StCDF1* accounts for the large effect QTL for foliage maturity on chromosome 5 (Kloosterman *et al*., 2013), tuber initiation is also influenced by other QTLs which possibly interact with the *earliness* locus on chromosome 5 (Van den Berg *et al*., 1996; Kittipadukal *et al*., 2012). *StCEN* was indeed identified in a genetic screen for inheritance of sprouting traits (Morris *et al*., 2019), hence evidencing that natural variation in this gene affects tuber initiation as well as tuber sprout growth. As alleles identified in the diploid parents of the cross encode identical proteins, the distinct function of these natural variants probably relies on their expression level (Morris *et al*., 2019). A more detailed study of these *StCEN* gene copies and the generation of molecular markers specific to these alleles will thus be of great assistance in breeding programmes aimed at the selection of potato genotypes with improved tuber life‐cycle characteristics.

## EXPERIMENTAL PROCEDURES

### Plant material and growth conditions

*Solanum tuberosum* cv. Desiree WT (Desiree WT) and *S. andigena* accession 7540 (ADG) WT plants used for transformation were propagated in 90 mm Petri dishes containing MS medium (Murashige and Skoog, 1962) supplemented with 20 g L^−1^ sucrose and 8 g L^−1^ agar at 18 ± 4°C, 16 h light, light intensity 100 μmol m^−2^ sec^−1^. Four‐week‐old *in vitro* subcultured plantlets were transferred to soil or hydroponic baths, and grown in the glasshouse, under controlled growth cabinet or hydroponic conditions. Glasshouse‐grown plants were transferred to 12 cm pots containing compost and grown under conditions of 16 h light (18°C) and 8 h dark (15°C). Light intensity ranged from 400 to 1000 μmol m^−2^ sec^−1^.

For cultivation in hydroponic growth baths (GT205 NFT Grow Tank, Progrow, https://www.pro‐grow.com/), plantlets were placed in rock‐wool plugs on 2.8 ml L^−1^ Hydro‐SuperMix liquid fertiliser (Bio Nova, Waalwijk, The Netherlands, https://www.bionovanutrients.com/), yielding 7% nitrogen (nitrate and ammonium nitrate), 4% phosphoric anhydride, 5% potassium oxide, 0.036% iron, 0.001% manganese and 0.004% zinc. This medium was continually pumped over the plant root system using a Micro‐Jet MC320 water pump (Progrow), and aerated using a ceramic airstone attached to an adjustable air pump (Hailea, https://www.hailea.com). Plants were fed with Hydro‐Supermix on a weekly basis and topped up with water daily to maintain the volume of the hydroponic system. Individual plants were visually scored every 3–4 days and a value assigned according to the stage of below‐ground development using the following scale: 0, developing stolons; 1, swelling stolons; 2, developing tubers (<1 cm); 3, tubers >1 cm.

The ADG plants used in the cabinet experiments were grown in the glasshouse under previously described conditions for 6 weeks and moved to growth cabinets under conditions of 8–16 h light (80% humidity) and 16–8 h dark (70% humidity), light intensity 300 μmol m^−2^ sec^−1^ and watered daily. Individual plants were visually scored for flower initiation every 7–10 days using the following scale: 0, no buds present; 2, early developing buds; 3, late developing buds; 4, open flowers.

### Generation of StCEN transgenic potato lines

The pB19‐35S‐CEN and pRNAi‐GG‐CEN constructs used in this study were described previously by Morris *et al*. (2019). For generation of the ADG transgenic lines, the pB19‐35S‐CEN and pRNAi‐GG‐CEN binary vectors were transformed into *Agrobacterium tumefaciens* strain AGL1 by electroporation, and cells were selected for their resistance to kanamycin and rifampicin. *Agrobacterium*‐mediated potato transformation was performed as described previously (Ducreux *et al*., 2005).

### RNA extraction and qRT‐PCR

Total RNA was extracted from potato leaf, stem, root, stolon and tuber samples using a RNeasy® Plant Mini Kit (Qiagen, https://www.qiagen.com/), following the manufacturer’s instructions. The first‐strand cDNA templates were generated by reverse transcription using a double‐primed RNA to cDNA EcoDry^™^ Premix kit (TaKaRa, Clontech, https://www.takarabio.com/about/our‐brands/clontech). Potato elongation factor 1‐alpha (*EF1α*) primers were used as a normalisation control (Nicot *et al*., 2005). The expression level of *StCEN* was determined using the StepOnePlus Real‐Time PCR system (Applied Biosystems, https://www.thermofisher.com/uk/en/home/brands/applied‐biosystems.html) and StepOne Software version 2.3 (Applied Biosystems). Gene‐specific primers and Universal Probe Library (UPL, Roche Life Science, https://lifescience.roche.com/) probes (Data S4) were used at a concentration of 0.2 µm and 0.1 µm, respectively. Thermal cycling conditions were: 10 min denaturation at 95°C followed by 40 cycles of 15 sec at 94°C and 60 sec at 60°C. Relative expression levels were calculated and the primers validated using the Delta–Delta Ct method (Livak and Schmittgen, 2001).

### Microarray analysis

A custom Agilent microarray (https://www.agilent.com/) designed to the predicted transcripts from assembly 3.4 of the DM potato genome was used as described (Hancock *et al*., 2014). Access to ordering the array design (AMADID 033033) from Agilent is available on request from the authors. The experimental design and complete datasets are available at ArrayExpress (E‐MTAB‐8346). Briefly, a single‐channel microarray design was utilised, with all swelling stolon RNA samples labelled with the Cy3 dye. A total of 18 microarrays were processed, consisting of three biological replicates of stolon material for each transgenic ADG line (RNAi and OE) and WT, from 23 and 38 day‐old plants grown under 12 h days. The RNA labelling and subsequent microarray processing was performed as described (Morris *et al*., 2014). Entire feature extraction (FE v.12.03.02, Agilent) datasets for each array were loaded as single‐channel data into GeneSpring software (v.7.3, Agilent) for further analysis. Data were normalised using default single‐channel settings: intensity values were set to a minimum of 0.01 and data from each array were normalised to the 50th percentile of all measurements on the array. Unreliable data, flagged as absent in all replicate samples by the FE software, were discarded. Statistical filtering of data to identify differentially expressed transcripts was achieved using volcano plots with thresholds of greater than twofold change and a Student’s *t*‐test value of *P* ≤ 0.05 applied. Clustering of gene expression profiles was performed in GeneSpring using the *k*‐means algorithm. Default parameters (100 iterations, Pearson measure as similarity correlation) were used to generate four cluster sets. Based on previous literature, a subset of genes that had already been associated with early tuberisation were selected for downstream analysis.

### Yeast two‐hybrid analysis

Constructs used for testing Y2H interaction between StCEN, StCEN‐m^RPFR^ mutant (bait) and the StFD and StFDL1a (prey) proteins were produced by LR recombination of pDONR201 Gateway® vectors containing full length ORFs into the pDEST32 and pDEST22 vectors (Invitrogen, https://www.thermofisher.com/us/en/home/brands/invitrogen) following the manufacturer’s instructions. The resulting constructs were transformed into the yeast strain MaV203 following the manufacturer’s standard protocol (Invitrogen). Bait and prey interactions were selected by growing transformed yeast cells on minimal synthetic dropout (SD)/–Leu/–Trp/–His selective medium containing 30 mm 3‐amino‐1,2,4‐triazole at 30°C for 2–3 days. The ability to grow on –His medium indicated a positive interaction.

### Bimolecular fluorescence complementation assays

Binary vectors for *in planta* expression of proteins as fusions to the C‐terminal of N‐terminal and C‐terminal portions of split‐YFP were produced by recombination of pDONR201 Gateway vectors into pCL112 and pCL113 (Bos *et al*., 2010). Recombinant *A. tumefaciens* strains bearing these constructs were grown with spectinomycin selection. Agroinfiltrations were performed as described elsewhere (Bos *et al*., 2010). Mixtures containing equal amounts of strains designed to express fusions to YN and YC were brought to final ODs between 0.03 and 1.0, depending on the experiment, and infiltrated into *N. benthamiana* leaves. Pairs of mixtures containing WT or mutant proteins were infiltrated into the opposing half‐leaves.

### Confocal imaging

Imaging was performed on a Zeiss LSM 710 microscope (Carl Zeiss Ltd, https://www.zeiss.com/) at 3 or 4 days after infiltration. A 514 nm laser was used for excitation. The YFP emission was collected between 515 and 550 nm and chlorophyll emission between 650 and 690 nm. The strength of the YFP signal was determined by collecting multiple stacks of images, near the epidermal cell layer, in a randomised pattern with a 10× objective lens. Fixed, subsaturating, collection conditions were used for test and control infiltrations from opposing half‐leaves (*n* ≥ 10 per set). The mean pixel intensity of maximum‐intensity projections was obtained using the Zen microscope software (Carl Zeiss Ltd). Pairwise, one‐tailed *t*‐tests were carried out in Microsoft Excel (https://www.microsoft.com/). Repetitions of experiments were carried out and all showed significant differences. Data for representative experiments are presented. That differences in signal strength were not a consequence of reduced accumulation of fusions to mutant proteins in comparison to fusions to WT proteins was confirmed through Western blotting of protein extracts from infiltrated leaves.

### Transient transactivation studies

For transactivation studies, the *StFD* and *StFDL1a* coding regions in pDONR201 were mobilised by LR Clonase II recombination into the pMDC7 vector (Curtis and Grossniklaus, 2003) to drive estradiol inducible expression of these factors. The *StCEN*/*TFL1* and *StSP6A* ORFs in the pDONR201 and pENTRY/D vectors were mobilised into the pGWB5 vector (Nakagawa *et al*., 2007) for 35S expression of the N‐terminal GFP fusions of these proteins. The *p6A*::LUC reporter was generated by amplifying a 2.64 kb region upstream of the start ATG of the PGSC0003DMT400060057 gene, using the *p6A_*for: 5′‐CACCTGTTAATTTCCTTTCTT‐3′ and *p6A*_rev: 5′‐CTCTAGGCTTGATAAAATTAAGT‐3′ primers. The PCR product was cloned into pENTRY/D TOPO and mobilised by LR Clonase II recombination into the pLuc‐Trap3 vector. The *pGERMIN3*::LUC reporter was generated by amplifying a 1.84 kb region upstream of the start ATG of the PGSC0003DMT400046995 gene, using the DMT46995prom*_*for: 5′‐AAAAAGCAGGCTTAATTCCCGCTGCCACTTTG‐3′ and DMT46995prom_rev: 5′‐AGAAAGCTGGGTAACAATTAGTTCAAACTCTGTG‐3′ primers. The PCR product was cloned into pDONR201 and mobilised by LR Clonase II recombination into the pLuc‐Trap3 vector.

For the luciferase assays, *N. benthamiana* leaves were co‐infiltrated with *Agrobacterium* strains expressing either the *p6A*::LUC or the *pGERMIN3*::LUC reporter and the StFDL1a, SP6A and StCEN/TFL1 effector constructs in the indicated combinations. Strains expressing the p19 silencing suppressor were added to the infiltration mixes to avoid silencing of these constructs. Bacterial cultures for the reporter constructs were used at an OD_600_ = 0.3, and the effector and p19 strains at OD_600_ = 0.7 and 1.0, respectively. To test activity of the reporter alone or the independent transcriptional control by each of the effectors, bacterial cultures were supplemented with an *Agrobacterium* strain expressing the empty pGWB5 vector to prevent differences in LUC activity due to competitive expression or dilution effects. Two days after infiltration, 0.6 cm diameter leaf discs were collected from the leaves and transferred to 96‐well microtitre plates containing 195 µl of 0.5× MS liquid medium, the d‐luciferin (Promega, https://www.promega.com) substrate (5 µg ml^−1^) and 10 µm estradiol to the induction of the StFD/StFDL1a effectors. Luminescence was measured with a LB 960 Microplate Luminometer (Berthold, https://www.berthold.com/). One disc was used per well and at least 12 disc replicates were measured per sample. The median of the activity values and standard error are represented.

## Author Contributions

XZ, RC, EMO and LD performed expression analysis and growth yield experiments. XZ, AGR and SC performed the protein interaction experiments. JS made the transgenic lines. SP performed the transactivation experiments. PH and JM performed the microarray experiments. MT, LT, GB and SP conceived the project and obtained funding for the work. MT, RC, XZ, AGR, SC and SP wrote the manuscript.

## Conflict of Interest

The authors have no conflicts of interest to declare.

## Supporting information


**Figure S1.** *StCEN* and *StSP6A* expression profiles during potato plant development.

**Figure S2.** Average tuber number per plant of ADG overexpression and RNA interference lines.

**Figure S3.** Clustering analysis of transcripts differentially expressed in stolons from ADG transgenic lines.

**Data S1.** List of genes that are associated with the four *k*‐means clusters shown in Figure S3.

**Data S2.** Microarray expression data of *StMADS* genes proposed as potential StSP6A targets.

**Data S3.** Quantification of yellow fluorescent protein fluorescence in bimolecular fluorescence complementation assays involving wild‐type and mutated interactors.

**Data S4.** Primer sequences used in this study.

## Data Availability

For the microarray experiment described in this paper, the experimental design and complete datasets are available at ArrayExpress (E‐MTAB‐8346).

## References

[tpj14898-bib-0001] Abe, M. , Kobayashi, Y. , Yamamoto, S. , Daimon, Y. , Yamaguchi, A. , Ikeda, Y. , Ichinoki, H. , Notaguchi, M. , Goto, K. and Araki, T. (2005) FD, a bZIP protein mediating signals from the floral pathway integrator FT at the shoot apex. Science, 309, 1052–1056. 16099979 10.1126/science.1115983

[tpj14898-bib-0002] Abelenda, J.A. , Navarro, C. and Prat, S. (2011) From the model to the crop: genes controlling tuber formation in potato. Curr. Opin. Biotechnol. 22, 287–292. 21168321 10.1016/j.copbio.2010.11.013

[tpj14898-bib-0003] Abelenda, J.A. , Navarro, C. and Prat, S. (2014) Flowering and tuberization: a tale of two nightshades. Trends Plant Sci. 19, 115–122. 24139978 10.1016/j.tplants.2013.09.010

[tpj14898-bib-0004] Abelenda, J.A. , Cruz‐Oró, E. , Franco‐Zorilla, J.M. and Prat, S. (2016) Potato StCONSTANS‐like1 suppresses storage organ formation by directly activating the FT‐like StSP5G repressor. Curr. Biol. 26, 872–881. 26972319 10.1016/j.cub.2016.01.066

[tpj14898-bib-0005] Adavi, Z. , Moradi, R. , Saeidnejad, A.H. , Tadayon, M.R. and Mansouri, H. (2018) Assessment of potato response to climate change and adaptation strategies. Sci. Hortic. 228, 91–102.

[tpj14898-bib-0006] Barman, A.R. and Banerjee, J. (2015) Versatility of germin‐like proteins in their sequences, expressions, and functions. Funct. Integr. Genomics, 15, 533–548. 26174051 10.1007/s10142-015-0454-z

[tpj14898-bib-0007] Bos, J.I. , Armstrong, M.R. , Gilroy, E.M. ***et al*** (2010) Phytophthora infestans effector AVR3a is essential for virulence and manipulates plant immunity by stabilizing host E3 ligase CMPG1. Proc. Natl. Acad. Sci. 107(21), 9909–9914. 20457921 10.1073/pnas.0914408107 PMC2906857

[tpj14898-bib-0008] Comadran, J. , Kilian, B. , Russell, J. ***et al*** (2012) Natural variation in a homolog of Antirrhinum CENTRORADIALIS contributed to spring growth habit and environmental adaptation in cultivated barley. Nat. Genet. 44, 1388. 23160098 10.1038/ng.2447

[tpj14898-bib-0009] Curtis, M.D. and Grossniklaus, U. (2003) A gateway cloning vector set for high‐throughput functional analysis of genes in planta. Plant Physiol. 133, 462–469. 14555774 10.1104/pp.103.027979 PMC523872

[tpj14898-bib-0010] Danilevskaya, O.N. , Meng, X. and Ananiev, E.V. (2010) Concerted modification of flowering time and inflorescence architecture by ectopic expression of TFL1‐like genes in maize. Plant Physiol. 153, 238–251. 20200067 10.1104/pp.110.154211 PMC2862429

[tpj14898-bib-0011] Ditta, G. , Pinyopich, A. , Robles, P. , Pelaz, S. and Yanofsky, M.F. (2004) The SEP4 gene of Arabidopsis thaliana functions in floral organ and meristem identity. Curr. Biol. 14(21), 1935–1940. 15530395 10.1016/j.cub.2004.10.028

[tpj14898-bib-0012] Ducreux, L.J. , Morris, W.L. , Taylor, M.A. and Millam, S. (2005) Agrobacterium‐mediated transformation of Solanum phureja. Plant Cell Rep. 24, 10–14. 15666166 10.1007/s00299-004-0902-z

[tpj14898-bib-0013] Eviatar‐Ribak, T. , Shalit‐Kaneh, A. , Chappell‐Maor, L. , Amsellem, Z. , Eshed, Y. and Lifschitz, E. (2013) A cytokinin‐activating enzyme promotes tuber formation in tomato. Curr. Biol. 23, 1057–1064. 23746638 10.1016/j.cub.2013.04.061

[tpj14898-bib-0014] Gao, H. , Wang, Z. , Li, S. , Hou, M. , Zhou, Y. , Zhao, Y. , Li, G. , Zhao, H. and Ma, H. (2018) Genome‐wide survey of potato MADS‐box genes reveals that StMADS1 and StMADS13 are putative downstream targets of tuberigen StSP6A. BMC Genom., 19, 726. 10.1186/s12864-018-5113-z PMC6171223 30285611

[tpj14898-bib-0015] Ghate, T.H. , Sharma, P. , Kondhare, K.R. , Hannapel, D.J. and Banerjee, A.K. (2017) The mobile RNAs, StBEL11 and StBEL29, suppress growth of tubers in potato. Plant Mol. Biol. 93, 563–578. 28084609 10.1007/s11103-016-0582-4

[tpj14898-bib-0016] Gutaker, R.M. , Weiß, C.L. , Ellis, D. , Anglin, N.L. , Knapp, S. , Fernández‐Alonso, J.L. , Prat, S. and Burbano, H.A. (2019) The origins and adaptation of European potatoes reconstructed from historical genomes. Nat. Ecol. Evol. 3, 1093–1101. 31235927 10.1038/s41559-019-0921-3

[tpj14898-bib-0017] Ham, B.K. , Li, G. , Kang, B.H. , Zeng, F. and Lucas, W.J. (2012) Overexpression of Arabidopsis plasmodesmata germin‐like proteins disrupts root growth and development. Plant Cell, 24, 3630–3648. 22960910 10.1105/tpc.112.101063 PMC3480292

[tpj14898-bib-0018] Hanano, S. and Goto, K. (2011) Arabidopsis TERMINAL FLOWER1 is involved in the regulation of flowering time and inflorescence development through transcriptional repression. Plant Cell, 23, 3172–3184. 21890645 10.1105/tpc.111.088641 PMC3203435

[tpj14898-bib-0019] Hancock, R.D. , Roberts, A.G. and Viola, R. (2008) A role for symplastic gating in the control of the potato tuber life cycle. Plant Signal. Behav. 3, 27–29. 19704762 10.4161/psb.3.1.4813 PMC2633952

[tpj14898-bib-0020] Hancock, R.D. , Morris, W.L. , Ducreux, L.J. ***et al*** (2014) Physiological, biochemical and molecular responses of the potato (*Solanum tuberosum* L.) plant to moderately elevated temperature. Plant Cell Environ. 37, 439–450. 23889235 10.1111/pce.12168

[tpj14898-bib-0021] Hannapel, D. and Banerjee, A. (2017) Multiple mobile mRNA signals regulate tuber development in potato. Plants, 6, 8. 10.3390/plants6010008 PMC5371767 28208608

[tpj14898-bib-0022] Hollwey, E. , Out, S. , Watson, M.R. , Heidmann, I. and Meyer, P. (2017) TET 3‐mediated demethylation in tomato activates expression of a CETS gene that stimulates vegetative growth. Plant Direct, 1, e00022. 31245668 10.1002/pld3.22 PMC6508569

[tpj14898-bib-0023] Horstman, A. , Tonaco, I.A.N. , Boutilier, K. and Immink, R.G.H. (2014) A cautionary note on the use of split‐YFP/BiFC in plant protein‐protein interaction studies. Int. J. Mol. Sci. 15, 9628–9643. 24886811 10.3390/ijms15069628 PMC4100113

[tpj14898-bib-0024] Iwata, H. , Gaston, A. , Remay, A. , Thouroude, T. , Jeauffre, J. , Kawamura, K. , Oyant, L.H. , Araki, T. , Denoyes, B. and Foucher, F. (2012) The TFL1 homologue KSN is a regulator of continuous flowering in rose and strawberry. Plant J. 69, 116–125. 21895811 10.1111/j.1365-313X.2011.04776.x

[tpj14898-bib-0025] Jaeger, K.E. , Pullen, N. , Lamzin, S. , Morris, R.J. and Wigge, P.A. (2013) Interlocking feedback loops govern the dynamic behavior of the floral transition in Arabidopsis. Plant Cell, 25, 820–833. 23543784 10.1105/tpc.113.109355 PMC3634691

[tpj14898-bib-0026] Kaneko‐Suzuki, M. , Kurihara‐Ishikawa, R. , Okushita‐Terakawa, C. , Kojima, C. , Nagano‐Fujiwara, M. , Ohki, I. , Tsuji, H. , Shimamoto, K. and Taoka, K.I. (2018) TFL1‐like proteins in rice antagonize rice FT‐like protein in inflorescence development by competition for complex formation with 14‐3‐3 and FD. Plant Cell Physiol. 59, 458–468. 29401229 10.1093/pcp/pcy021

[tpj14898-bib-0027] Kittipadukal, P. , Bethke, P.C. and Jansky, S.H. (2012) The effect of photoperiod on tuberization in cultivated× wild potato species hybrids. Potato Res. 55, 27–40.

[tpj14898-bib-0028] Kloosterman, B. , Abelenda, J.A. , Gomez, M. del M.C. ***et al*** (2013) Naturally occurring allele diversity allows potato cultivation in northern latitudes. Nature, 495, 246–250. 23467094 10.1038/nature11912

[tpj14898-bib-0029] Kwak, M. , Toro, O. , Debouck, D.G. and Gepts, P. (2012) Multiple origins of the determinate growth habit in domesticated common bean (Phaseolus vulgaris). Ann. Bot. 110, 1573–1580. 23019270 10.1093/aob/mcs207 PMC3503494

[tpj14898-bib-0030] Lehretz, G.G. , Sonnewald, S. , Hornyik, C. , Corral, J.M. and Sonnewald, U. (2019) Post‐transcriptional Regulation of FLOWERING LOCUS T modulates heat‐dependent source‐sink development in potato. Curr. Biol. 29, 1614–1624. 31056391 10.1016/j.cub.2019.04.027

[tpj14898-bib-0031] Livak, K.J. and Schmittgen, T.D. (2001) Analysis of relative gene expression data using real‐time quantitative PCR and the 2*−ΔΔCT* method. Methods, 25, 402–408. 11846609 10.1006/meth.2001.1262

[tpj14898-bib-0032] Mahajan, A. , Bhogale, S. , Kang, I.H. , Hannapel, D.J. and Banerjee, A.K. (2012) The mRNA of a Knotted1‐like transcription factor of potato is phloem mobile. Plant Mol. Biol. 79, 595–608. 22638904 10.1007/s11103-012-9931-0

[tpj14898-bib-0033] Martin, A. , Adam, H. , Díaz‐Mendoza, M. , Żurczak, M. , González‐Schain, N.D. and Suárez‐López, P. (2009) Graft‐transmissible induction of potato tuberization by the microRNA miR172. Development, 136, 2873–2881. 19666819 10.1242/dev.031658

[tpj14898-bib-0034] Moraes, T.S. , Dornelas, M.C. and Martinelli, A.P. (2019) FT/TFL1: calibrating plant architecture. Front. Plant Sci. 10, 97. 30815003 10.3389/fpls.2019.00097 PMC6381015

[tpj14898-bib-0035] Morris, W.L. , Hancock, R.D. , Ducreux, L.J.M. ***et al*** (2014) Day length dependent restructuring of the leaf transcriptome and metabolome in potato genotypes with contrasting tuberization phenotypes. Plant Cell Environ. 37, 1351–1363. 24236539 10.1111/pce.12238

[tpj14898-bib-0036] Morris, W.L. , Alamar, M.C. , Lopez‐Cobollo, R.M. ***et al*** (2019) A member of the TERMINAL FLOWER1/CENTRORADIALIS gene family controls sprout growth in potato tubers. J. Exp. Bot. 70, 835–843. 30395257 10.1093/jxb/ery387 PMC6363080

[tpj14898-bib-0037] Murashige, T. and Skoog, F. (1962) A revised medium for rapid growth and bio‐assays with tobacco tissue cultures. Physiol. Plant. 15, 473–497.

[tpj14898-bib-0038] Nakagawa, T. , Kurose, T. , Hino, T. , Tanaka, K. , Kawamukai, M. , Niwa, T.K. , Matsuoka, K. , Jinbo, T. and Kimura, T. (2007) Development of series of gateway binary vectors, pGWBs, for realizing efficient construction of fusion genes for plant transformation. J. Biosci. Bioeng. 104, 34–41. 17697981 10.1263/jbb.104.34

[tpj14898-bib-0039] Navarro, C. , Abelenda, J.A. , Cruz‐Oró, E. , Cuéllar, C.A. , Tamaki, S. , Silva, J. , Shimamoto, K. and Prat, S. (2011) Control of flowering and storage organ formation in potato by FLOWERING LOCUS T. Nature, 478, 119–122. 21947007 10.1038/nature10431

[tpj14898-bib-0040] Nicot, N. , Hausman, J.F. , Hoffmann, L. and Evers, D. (2005) Housekeeping gene selection for real‐time RT‐PCR normalization in potato during biotic and abiotic stress. J. Exp. Bot. 56, 2907–2914. 16188960 10.1093/jxb/eri285

[tpj14898-bib-0041] Plantenga, F.D. , Bergonzi, S. , Abelenda, J.A. , Bachem, C.W. , Visser, R.G. , Heuvelink, E. and Marcelis, L.F. (2018) The tuberization signal StSP6A represses flower bud development in potato. J. Exp. Bot. 70, 937–948. 10.1093/jxb/ery420 30481308

[tpj14898-bib-0042] Pnueli, L. , Carmel‐Goren, L. , Hareven, D. , Gutfinger, T. , Alvarez, J. , Ganal, M. , Zamir, D. and Lifschitz, E. (1998) The SELF‐PRUNING gene of tomato regulates vegetative to reproductive switching of sympodial meristems and is the ortholog of CEN and TFL1. Development, 125, 1979–1989. 9570763 10.1242/dev.125.11.1979

[tpj14898-bib-0050] Potato Genome Sequencing Consortium . (2011) Genome sequence and analysis of the tuber crop potato. Nature, 475(7355), 189. 21743474 10.1038/nature10158

[tpj14898-bib-0043] Rodriguez‐Falcon, M. , Bou, J. and Prat, S. (2006) Seasonal control of tuberization in potato: conserved elements with the flowering response. Annu. Rev. Plant Biol. 57, 151–180. 16669759 10.1146/annurev.arplant.57.032905.105224

[tpj14898-bib-0044] Teo, C.J. , Takahashi, K. , Shimizu, K. , Shimamoto, K. and Taoka, K.I. (2016) Potato tuber induction is regulated by interactions between components of a tuberigen complex. Plant Cell Physiol. 58, 365–374. 10.1093/pcp/pcw197 28028166

[tpj14898-bib-0045] Van den Berg, J.H. , Ewing, E.E. , Plaisted, R.L. , McMurry, S. and Bonierbale, M.W. (1996) QTL analysis of potato tuberization. Theor. Appl. Genet. 93, 307–316. 24162285 10.1007/BF00223170

[tpj14898-bib-0046] Varkonyi‐Gasic, E. , Wang, T. , Voogd, C. , Jeon, S. , Drummond, R.S. , Gleave, A.P. and Allan, A.C. (2019) Mutagenesis of kiwifruit CENTRORADIALIS‐like genes transforms a climbing woody perennial with long juvenility and axillary flowering into a compact plant with rapid terminal flowering. Plant Biotechnol. J., 17(5), 869–880. 30302894 10.1111/pbi.13021 PMC6587708

[tpj14898-bib-0047] Viola, R. , Roberts, A.G. , Haupt, S. , Gazzani, S. , Hancock, R.D. , Marmiroli, N. , Machray, G.C. and Oparka, K.J. (2001) Tuberization in potato involves a switch from apoplastic to symplastic phloem unloading. Plant Cell, 13, 385–398. 11226192 10.1105/tpc.13.2.385 PMC102249

[tpj14898-bib-0048] Wigge, P.A. , Kim, M.C. , Jaeger, K.E. , Busch, W. , Schmid, M. , Lohmann, J.U. and Weigel, D. (2005) Integration of spatial and temporal information during floral induction in Arabidopsis. Science, 309, 1056–1059. 16099980 10.1126/science.1114358

[tpj14898-bib-0049] Zhou, J. , Fang, H. , Shan, J. , Gao, X. , Chen, L. , Xie, C. , Xie, T. and Liu, J. (2014) A major QTL located on chromosome V associates with in vitro tuberization in a tetraploid potato population. Mol. Genet. Genomics, 289, 575–587. 24619101 10.1007/s00438-014-0832-6

