# Supplementary figures and images for: TERMINAL FLOWER‐1/CENTRORADIALIS inhibits tuberisation via protein interaction with the tuberigen activation complex
Source: Plant J. 2020 Jul 14;103(6):2263–78. doi: 10.1111/tpj.14898 (PMC7540344; doi:10.1111/tpj.14898)

(a)

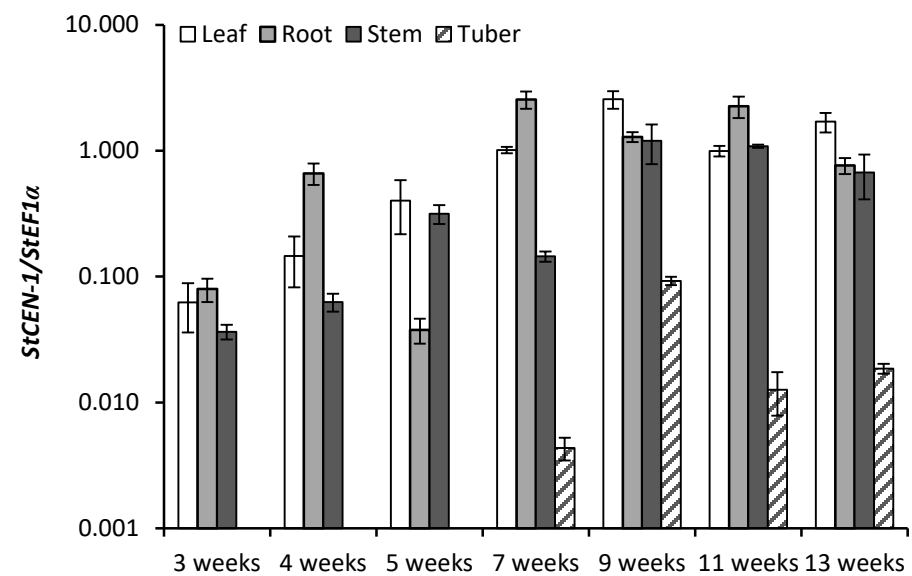

(b)

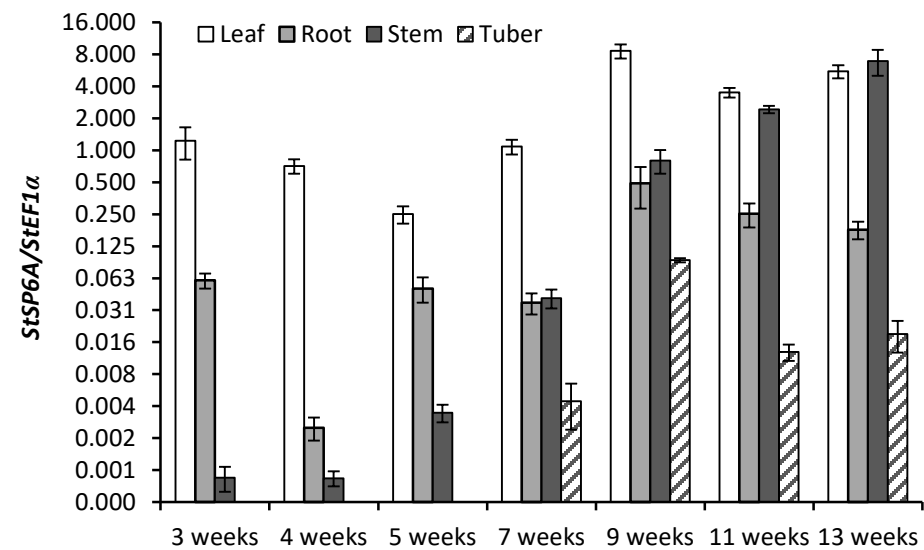

Supplement: Supplementary file 1 — Figure S1. StCEN and StSP6A expression profiles during potato plant development. [file TPJ-103-2263-s001.pdf]

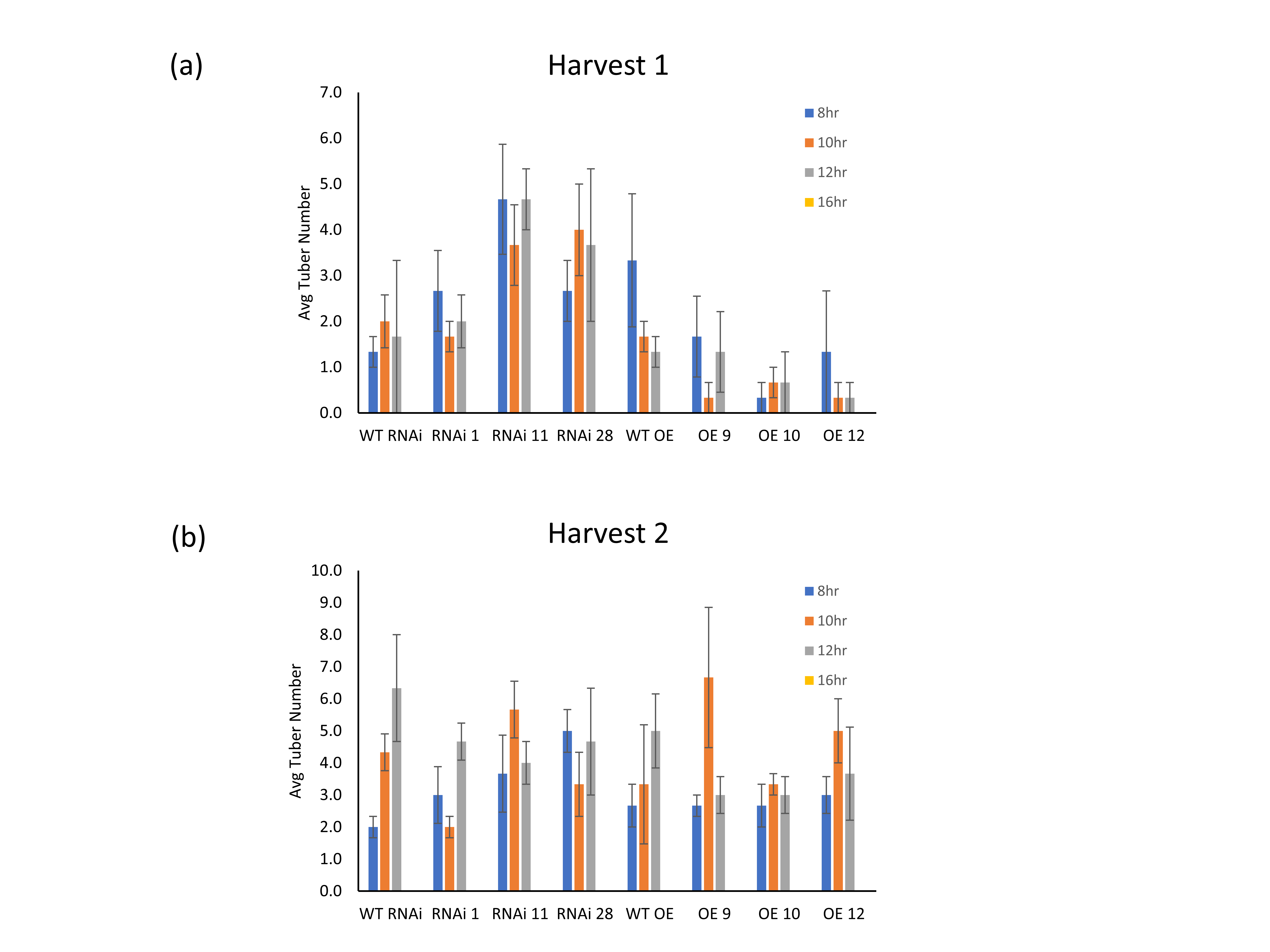

Supplement: Supplementary file 2 — Figure S2. Average tuber number per plant of ADG overexpression and RNA interference lines. [file TPJ-103-2263-s002.tif]

Set 1

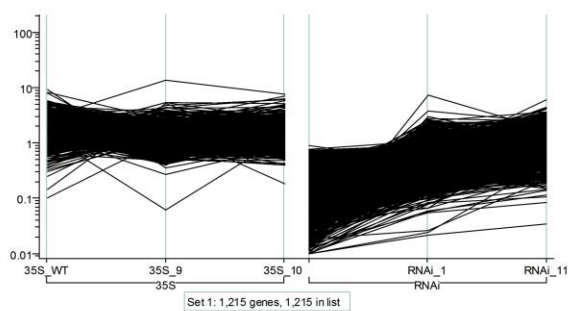

Set 2

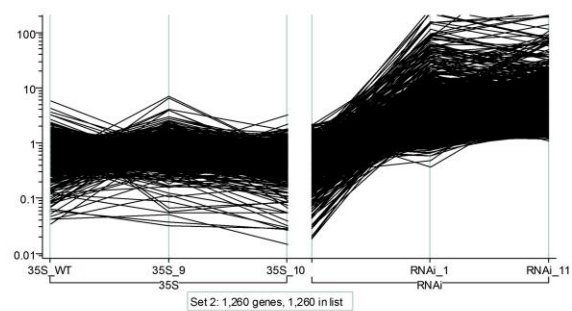

Set 3

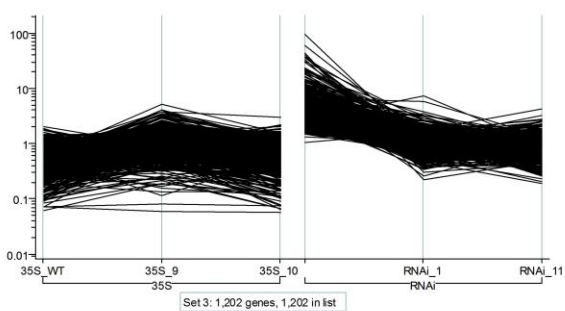

Set 4

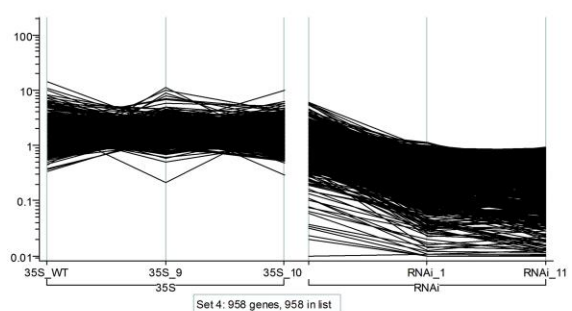

Supplement: Supplementary file 3 — Figure S3. Clustering analysis of transcripts differentially expressed in stolons from ADG transgenic lines. [file TPJ-103-2263-s003.pdf]
