# Supplementary material for: TERMINAL FLOWER‐1/CENTRORADIALIS inhibits tuberisation via protein interaction with the tuberigen activation complex
Source: Plant J. 2020 Jul 14;103(6):2263–78. doi: 10.1111/tpj.14898 (PMC7540344; doi:10.1111/tpj.14898)
Supplement: Supplementary file 5 — Data S2. Microarray expression data of StMADS genes proposed as potential StSP6A targets. [file TPJ-103-2263-s005.docx]

**Supplemental Dataset 2:** Microarray expression data of *StMADS* genes proposed as potential StSP6A targets. Values presented are the average fold change value of 3 independent biological replicates compared with the WT control. Asterisks denote values significantly different between transgenic lines and wild type controls as determined by Student’s T-test (P<0.05) and volcano plot filtering.

|  | **StMADS 1** | **StMADS 3** | **StMADS 11** | **StMADS 12** | **StMADS 13** | **StMADS 17** | **StMADS 27** |
| --- | --- | --- | --- | --- | --- | --- | --- |
| **OE_9** | 0.71 | 0.15* | 1.03 | 0.16 | 0.44 | 0.72 | 1.84 |
| **OE_10** | 0.98 | 0.25* | 0.72 | 0.35 | 0.72 | 0.98 | 0.74 |
| **RNAi_1** | 0.86 | 0.22* | 0.37* | 0.85 | 1.36 | 4.02* | 0.89 |
| **RNAi_11** | 1.20 | 0.72 | 0.61 | 0.94 | 1.83 | 4.28* | 2.09 |
