# Supplementary material for: TERMINAL FLOWER‐1/CENTRORADIALIS inhibits tuberisation via protein interaction with the tuberigen activation complex
Source: Plant J. 2020 Jul 14;103(6):2263–78. doi: 10.1111/tpj.14898 (PMC7540344; doi:10.1111/tpj.14898)
Supplement: Supplementary file 6 — Data S3. Quantification of yellow fluorescent protein fluorescence in bimolecular fluorescence complementation assays involving wild‐type and mutated interactors. [file TPJ-103-2263-s006.docx]

**Supplemental Dataset 3.** **Quantification of YFP fluorescence in BiFC assays involving wild-type and mutated interactors.**

Mean represents the mean of mean pixel intensity values in the YFP channel for maximum intensity projections of image stacks collected using a randomized method from infiltrated areas +/- standard error (n ≥ 10). Whether mutated proteins gave reduced signals was tested using a one-tailed, t-test comparing data from pairs of infiltrations containing wild-type or mutant protein fusions on opposing half-leaves.

| Pairs | YN fusion | YC fusion | Mean +/- SE | P |
| --- | --- | --- | --- | --- |
| 1 | StCEN1_WT_ | FD | 1.480 +/- 0.083 | <0.000001 |
|  | StCEN1_mRFPR_ | FD | 0.551 +/- 0.027 |  |
| 2 | StCEN1_WT_ | FDL1a | 1.417 +/- 0.083 | <0.000001 |
|  | StCEN1_mRFPR_ | FDL1a | 0.504 +/- 0.019 |  |
| 3 | StCEN1_WT_ | 14-3-3a | 3.715 +/- 0.170 | <0.005 |
|  | StCEN1_mRFPR_ | 14-3-3a | 2.763 +/- 0.243 |  |
| 4 | StCEN1_WT_ | 14-3-3f | 6.131 +/- 0.326 | <0.005 |
|  | StCEN1_mRFPR_ | 14-3-3f | 4.923 +/- 0.237 |  |
| 5 | FDL1a_WT_ | StCEN1 | 1.865 +/- 0.104 | <0.0000001 |
|  | FDL1a_mT224A_ | StCEN1 | 0.546 +/- 0.029 |  |
