# Supplementary material for: TERMINAL FLOWER‐1/CENTRORADIALIS inhibits tuberisation via protein interaction with the tuberigen activation complex
Source: Plant J. 2020 Jul 14;103(6):2263–78. doi: 10.1111/tpj.14898 (PMC7540344; doi:10.1111/tpj.14898)
Supplement: Supplementary file 7 — Data S4. Primer sequences used in this study. [file TPJ-103-2263-s007.docx]

| **Supplementary Dataset 4.** Primers sequences used in this study | | | |  |  |
| --- | --- | --- | --- | --- | --- |
|  | PGSC/ITAG ID | Purpose | Forward | Reverse | Probe number |
|  |  |  |  |  |  |
| *StEF1a* | PGSC0003DMT400059830 | QPCR | CTTGACGCTCTTGACCAGATT | GAAGACGGAGGGGTTTGTCT | 113 |
| *StCEN* | PGSC0003DMT400037143 | QPCR OF OEX | TGGGAGCAAACAAGTTTCTAATG | TTCGACACGAGGTTGAGTTG | 143 |
| *StCEN* | PGSC0003DMT400037143 | QPCR OF RNAi | AATGCCCAGAGAGAAACTGC | ATTTTGTGTGTGTGTGTGTCAAAT | 40 |
| *StSP6A A1* | PGSC0003DMT400060057 | QPCR | GGACGATCTTCGCAACTTTT | TCAAGTCAGGGTTGCTTGG | 138 |
| *StSP5G* | sotub05g026750.1.1 | QPCR | TGGAGATGATCTTCGCACTTT | TTGCTAGGGTTTGGAGCATC | 138 |
| *StCEN* | PGSC0003DMT400037143 | Cloning of OEX | AAGTCGACAAAAATGTCTTCTAGAGGTACTTG | AAGTCGACAAATTCATCTTCTTCTAGCTGCAG |  |
| *StCEN* | PGSC0003DMT400037143 | Cloning of RNAi | ACCAGGTGTCAGGAGCCACTTGCAGTAGGGAGAGT | ACCAGGTCTCATCGTCCAATCCATTTTCCGCCGAA |  |
